# Transcriptome Analysis of Cyclooctasulfur Oxidation and Reduction by the Neutrophilic Chemolithoautotrophic *Sulfurovum indicum* from Deep-Sea Hydrothermal Ecosystems

**DOI:** 10.3390/antiox12030627

**Published:** 2023-03-03

**Authors:** Shasha Wang, Lijing Jiang, Liang Cui, Karine Alain, Shaobin Xie, Zongze Shao

**Affiliations:** 1 Key Laboratory of Marine Genetic Resources, Third Institute of Oceanography, Ministry of Natural Resources, Xiamen 361005, China; 2 State Key Laboratory Breeding Base of Marine Genetic Resources, Key Laboratory of Marine Genetic Resources of Fujian Province, Xiamen 361005, China; 3 Sino-French Laboratory of Deep-Sea Microbiology (MicrobSea), Xiamen 361005, China; 4 Department of Bioengineering and Biotechnology, Huaqiao University, Xiamen 361021, China; 5 CNRS, Université Brest, Ifremer, Unité Biologie et Ecologie des Ecosystèmes Marins Profonds BEEP, UMR 6197, IRP 1211 MicrobSea, IUEM, Rue Dumont d’Urville, F-29280 Plouzané, France; 6 Southern Marine Science and Engineering Guangdong Laboratory (Zhuhai), Zhuhai 519000, China

**Keywords:** *Campylobacterota*, *Sulfurovum*, sulfur oxidation, sulfur reduction, cyclooctasulfur activation

## Abstract

Chemolithoautotrophic *Campylobacterota* are widespread and predominant in worldwide hydrothermal vents, and they are key players in the turnover of zero-valence sulfur. However, at present, the mechanism of cyclooctasulfur activation and catabolism in *Campylobacterota* bacteria is not clearly understood. Here, we investigated these processes in a hydrothermal vent isolate named *Sulfurovum indicum* ST-419. A transcriptome analysis revealed that multiple genes related to biofilm formation were highly expressed during both sulfur oxidation and reduction. Additionally, biofilms containing cells and EPS coated on sulfur particles were observed by SEM, suggesting that biofilm formation may be involved in S^0^ activation in *Sulfurovum* species. Meanwhile, several genes encoding the outer membrane proteins of OprD family were also highly expressed, and among them, gene IMZ28_RS00565 exhibited significantly high expressions by 2.53- and 7.63-fold changes under both conditions, respectively, which may play a role in sulfur uptake. However, other mechanisms could be involved in sulfur activation and uptake, as experiments with dialysis bags showed that direct contact between cells and sulfur particles was not mandatory for sulfur reduction activity, whereas cell growth via sulfur oxidation did require direct contact. This indirect reaction could be ascribed to the role of H_2_S and/or other thiol-containing compounds, such as cysteine and GSH, which could be produced in the culture medium during sulfur reduction. In the periplasm, the sulfur-oxidation-multienzyme complexes *soxABXY_1_Z_1_* and *soxCDY_2_Z_2_* are likely responsible for thiosulfate oxidation and S^0^ oxidation, respectively. In addition, among the four *psr* gene clusters encoding polysulfide reductases, only *psrA_3_B_3_C_3_* was significantly upregulated under the sulfur reduction condition, implying its essential role in sulfur reduction. These results expand our understanding of the interactions of *Campylobacterota* with the zero-valence sulfur and their adaptability to deep-sea hydrothermal environments.

## 1. Introduction

Sulfur occurs in many oxidation states and is of central importance in biogeochemical cycles [1,2]. As an essential component of biomass, sulfur is assimilated into organic compounds and also participates in energy-yielding processes as an electron acceptor or donor in chemolithautotrophic, photolithoautotrophic and heterotrophic microorganisms [3,4,5,6]. Elemental sulfur is present in a variety of environments, including marine sediments, microbial mats, cold/hot springs, oxygen-minimum zones, glacial shields, volcanic soils and hydrothermal vents, where it always accumulates in amounts visible to the naked eye [7,8,9,10,11,12]. In these cases, sulfur occurs mostly in the form of zero-valence sulfur (S^0^), with cyclooctasulfur (S_8_) as the most stable and common form [13].

As elemental sulfur is extremely poorly soluble in water [14], an initial activation reaction, by direct or indirect interaction between sulfur and cells, is required prior to its uptake [15]. In acidophilic sulfur-oxidizing bacteria, a direct contact between cells and sulfur is necessary [16,17]. For example, in members of the genera *Acidithiobacillus* and *Acidiphilium*, extracellular S^0^ is activated by reacting with reactive thiol groups (-SH) of special outer membrane proteins, forming -S*_n_*H (*n* ≥ 2) complexes and then transporting it to the periplasm, where it is oxidized [18,19]. Extracellular polymers (e.g., exopolysaccharides EPS), cell surface proteins, extracellular vesicles and flagella play a key role in the attachment of bacterial cells to the S^0^ surface during this process [20,21,22]. In some sulfur-reducing microorganisms such as *Acidilobus sulfurireducens* 18D70 and *Desulfurella amilsii* TR1, the physical contact of cells and S^0^ is not mandatory [23,24]. These indirect interactions could occur through the formation of polysulfides generated by the nucleophilic attack on sulfur by sulfide, or through extracellular electron transport via pili or redox cofactors present in biofilms [25].

When S^0^ is activated and transported into the cell, it is catalyzed by different enzymes for oxidation or reduction to supply energy for growth. The bacterial oxidation of S^0^ has been extensively studied in various sulfur-oxidizing bacteria [26,27,28]. In the periplasmic space, various enzymes, such as sulfur-oxidizing-multienzyme complex (Sox), tetrathionate hydrolase (TetH) and thiosulfate dehydrogenase (Tsd), participate in the further oxidation of thiol-bound sulfane sulfur atoms (R-S-S_n_H) [17,29]. The Sox pathway, which is governed by the conserved operon SoxXYZABCD, operates in photo- and chemo-lithotrophic *Alphaproteobacteria* and *Campylobacterota* [7,30]. This enzymatic complex includes the c-type cytochromes SoxAX, the sulfur-compound-binding module SoxYZ, the sulfate thiol esterase SoxB, and a sulfur dehydrogenase Sox(CD)_2_ [31]. In the cytoplasmic space, sulfur can be oxidized to sulfite by a series of enzymes, such as sulfur oxygenase reductase (Sor), thiosulfate sulfurtransferase (Tst), heterodisulfide reductase (Hdr) and sulfur dioxygenase (Sdo) [19,27]. For the reduction of sulfur, three main enzymes including polysulfide reductase (Psr), sulfur reductase (Sre) and sulfide dehydrogenase (Sudh) have been purified and characterized in a limited number of S^0^ reducers, such as *Wolinella succinogenes*, *Pyrococcus furiosus*, *Acidianus ambivalens* and members of the *Desulfurellaceae* family [24,32,33]. The membrane-bound polysulfide reductase consists of three subunits (PsrABC). PsrA is the catalytic subunit of the Psr protein for polysulfide reduction to sulfide. PsrB on the periplasmic side of the membrane contains several [Fe-S] clusters, which likely mediate the electron transfer from the membrane anchor (PsrC) to the catalytic subunit (PsrA) of the Psr [15]. Psr contains menaquinone, which could serve as an electron acceptor for the hydrogenase, and could also be an electron donor for polysulfide/S^0^ reduction [32]. Our recent work revealed that two sulfur-reduction pathways performed by periplasmic and cytoplasmic Psr coexisted in chemolithoautotrophic *Sulfurimonas* from deep-sea hydrothermal vents, coupled with different energy conservation pathways [34].

The phylum *Campylobacterota* (formerly *Epsilonproteobacteria*) includes representatives that are primary producers of hydrothermal ecosystems and accounts for 66–98% of the microorganisms associated with the substrates of some hydrothermal vents [35,36,37]. The genus *Sulfurovum*, which belongs to *Campylobacterota*, was first isolated and identified in 2004 from deep-sea hydrothermal vent sediments [38]. It is widely distributed in sulfur-rich marine environments and is very abundant in the fluids of deep-sea hydrothermal vents worldwide [39,40]. In particular, members of the genus *Sulfurovum* are frequently found as endo- and/or epi-symbionts of various deep-sea hydrothermal vent animals [41]. It also has been suggested that *Sulfurovum* and *Sulfurimonas* species especially occupy the niche of S^0^/S_8_ oxidation in marine sediments and in hydrothermal vents [6,42]. All these observations tend to indicate that *Sulfurovum* taxa play an important ecological role in deep-sea hydrothermal vents. Furthermore, the isolates of *Sulfurovum* identified to date generally use H_2_ or reduced-sulfur compounds as electron donors, and oxygen, nitrate or elemental sulfur as terminal electron acceptors [38,43].

In our previous study, a deep-sea bacterium designated *Sulfurovum indicum* ST-419 was isolated from a hydrothermal plume on the Wocan-1 hydrothermal site of the northwestern Indian Ocean [43]. Wocan-1 on the Carlsberg Ridge (60°68′ E, 6°56′ N) was discovered in March 2017 during the COMRA DY 38 oceanic scientific cruise [43]. Interestingly, this strain can grow vigorously with S^0^ as the sole electron donor (S^0^ oxidation), as well as the terminal electron acceptor (S^0^ reduction). Sulfur-related energy metabolism in *Sulfurovum* is particularly important for issues related to the ecology and biogeochemistry of deep-sea hydrothermal ecosystems. In this study, we aimed to elucidate the mechanisms involved in the activation and transport of cylcooctasulfur, in addition to the S^0^ oxidation and reduction pathways. Understanding the interaction of *Sulfurovum* with cylcooctasulfur will help us gain insight into the role of *Campylobacterota* in various ecological niches.

## 2. Materials and Methods

### 2.1. Bacterial Strains and Growth Conditions

Cultures of strain ST-419 were routinely grown in a modified MMJS medium at 37 ℃ as previously described [35]. For sulfur oxidation cultures, either elemental sulfur (5%, *w/v*) or thiosulfate (10 mM) was added as the sole electron donor, and nitrate (10 mM) as the sole electron acceptor. In both cases, the headspace of the anaerobic bottles was filled with 80% N_2_/20% CO_2_ (200 kPa). For sulfur reduction cultures, S^0^ (5%, *w/v*) was added as the sole electron acceptor with molecular hydrogen as the sole electron donor in anaerobic bottles filled with a gas phase of 80% H_2_/20% CO_2_ (200 kPa). As a control of sulfur reduction, nitrate reduction was set in parallel, with nitrate (10 mM) as the sole electron acceptor under a gas phase of 80% H_2_/20% CO_2_ (200 kPa). S^0^ granules were heat-treated prior to use by incubation at 95 °C for 2 h, then stored at room temperature [34]. Bacterial growth was monitored by cell counting over the incubation using a phase-contrast microscope (Eclipse 80i, Nikon, Tokyo, Japan). The anaerobic bottles under S^0^ oxidation and reduction were submerged in an ultrapure water solution and sonicated (WVR Ultrasonic cleaner, 80 W) for 1 min. All cultivation tests were performed in triplicate. Growth rates (µ, h^−1^) were calculated at multiple time points on replicate cultures and averaged using: µ = (lnN_2_ − lnN_1_)/(t_2_ − t_1_), where N_2_ and N_1_ represent the number of cells mL^−1^ at time (hours of incubation) t_2_ and t_1_, respectively. Generation times (tg; measured in hours) were calculated using: tg = (ln2)/µ [44]. The morphology and size of the cells were observed with a transmission electron microscopy (Model JEM-1230; JEOL, Tokyo, Japan).

### 2.2. Chemical Analyses

For direct analysis, samples were taken from cultures with N_2_-flushed syringes. Concentrations of thiosulfate, sulfate and nitrate were measured after filtration (0.2 µm, cellulose-acetate) using ion chromatography (ICS-2100, Thermo Scientific, Waltham, MA, USA) [45]. The concentrations of hydrogen and nitrogen in the headspace of the bottles were determined by gas chromatography (CP-2002, GL-Science, Tokyo, Japan) [46]. Dissolved sulfide concentrations were determined using the methylene blue assay as previously described [47]. All cultivation tests were performed in triplicate.

### 2.3. Transcriptome Analysis

Cells of strain ST-419 cultivated with different electron donors and acceptors under four types of catabolic conditions including S^0^ oxidation, thiosulfate oxidation, S^0^ reduction and nitrate reduction were harvested during the exponential growth phase, and centrifuged at 8000 rpm for 10 min at 4 °C. Resulting bacterial pellets obtained were rapidly frozen in liquid nitrogen and used for total RNA extraction. Transcriptome analysis was performed by Novogene (Tianjin, China). Detailed protocols of these procedures, including library preparation, clustering and sequencing, and the data analyses, are described in the Supplementary Materials. RNA-Seq reads were deposited in GenBank under accession numbers SRR22829317 to SRR22829328.

### 2.4. Quantitative Real-Time PCR Analyses

Fifteen genes were chosen for verification of RNA-Seq data by qRT-PCR. Total RNAs from the four culture conditions described above were extracted using TRIzol^®^ reagent (Invitrogen, Waltham, MA, USA) and were then reverse-transcribed into cDNA using PremixScript™ RT reagent Kit (Takara, Otsu, Shiga, Japan). RNA degradation and contamination were monitored on 1% agarose gels. RNA purity and concentration were checked using the NanoPhotometer^®^ spectrophotometer (IMPLEN, Calabasas, CA, USA). The expression levels of the 15 selected genes were determined using SYBR^®^ Premix Ex Taq™ (RR420, Takara, Japan) with a Light cycler^®^480 (Roche, Switzerland) according to the manufacturer’s recommendations. After the cycling protocol, a melting curve was generated in order to detect single-gene-specific peaks and to check for the presence of primer–dimer peaks. The amplification efficiency was analyzed as follows: E = 10(^−1/Slope^) – 1, and amplification efficiencies were approximately ranged from 98.86 to 99.99%. The 16S rRNA gene was used as an internal reference, and the relative gene expression was calculated using the 2^−ΔΔCt^ method [48]. Specific primers for the 15 genes and 16S rRNA were designed using Primer 6.0 as shown in Table S1. Three independent biological replicates were performed for each condition, and three technical replicates were performed for each reaction.

### 2.5. Experiment with Dialysis Membranes to Test Cell Contact with S^0^ Granules

Elemental sulfur was enclosed in dialysis membranes in batch cultures to examine the need for physical contact between cells and bulk-solid sulfur during the S^0^ oxidation and reduction processes. S^0^ was added to dialysis membranes (Spectrum Laboratories, USA) with pore sizes of 6 to 8 kDa and 12 to 14 kDa, and then secured with dialysis clips. Before use, all dialysis membranes were incubated at 80 °C in sterile deionized water for 24 h to remove preservatives, and this process was repeated three times, with the deionized water being replaced each time [49]. Cultures grown with elemental sulfur exposed fully to the medium (no dialysis membrane) were used as positive controls. Uninoculated media containing sulfur that was sequestered in dialysis membranes were used as negative controls. The sulfide and sulfate production, as well as the cell density, were monitored as described above. All of the treatments were performed in triplicate.

### 2.6. Sequence and Phylogenetic Analyses

The gene clusters encoding proteins involved in polysulfide reductases were identified by a local BLASTP protein similarity search using previously characterized gene sequences (e.g., polysulfide reductase from *W. Succinogenes* and *Sulfurovum* sp. NBC37-1) or by ontology using functional search terms (e.g., sulfur, polysulfide). For the BLASTP analysis, we used an amino acid similarity cutoff of >30%, alignment coverage > 80% and an e-value cutoff of 1E-5. Sequences were then aligned using ClustalW [50] and viewed in an on-site program (http://www.bio-soft.net/sms/index.html, accessed on 17 August 2022). A phylogenetic tree was reconstructed by the maximum-likelihood method using RAxML version 8.2.11 with the GTR + CAT model [51]. PSI-BLAST and DELTA-BLAST [52] were used for the domain analysis of outer membrane proteins. BOCTOPUS (boctopus.bioinfo.se) and PRED-TMMB (http://bioinformatics.biol.uoa.gr////PRED-TMBB, accessed on 12 October 2022) were used to search for beta-barrel topology. Signal peptides were predicted using SignalP 4.0 (http://www.cbs.dtu.dk/services/SignalP/, accessed on 26 October 2022).

### 2.7. Statistical Analysis

The significant differences among groups were subjected to one-way analysis of variance (one-way ANOVA) and multiple comparisons using the SPSS 19.0 program. A statistical significance was defined in our study by *p* < 0.05 (indicated by * in all figures) or *p* < 0.01 (indicated by ** in all figures).

## 3. Results

### 3.1. Growth Kinetics under Two Different Sulfur Oxidation Conditions

**Figure 1:**
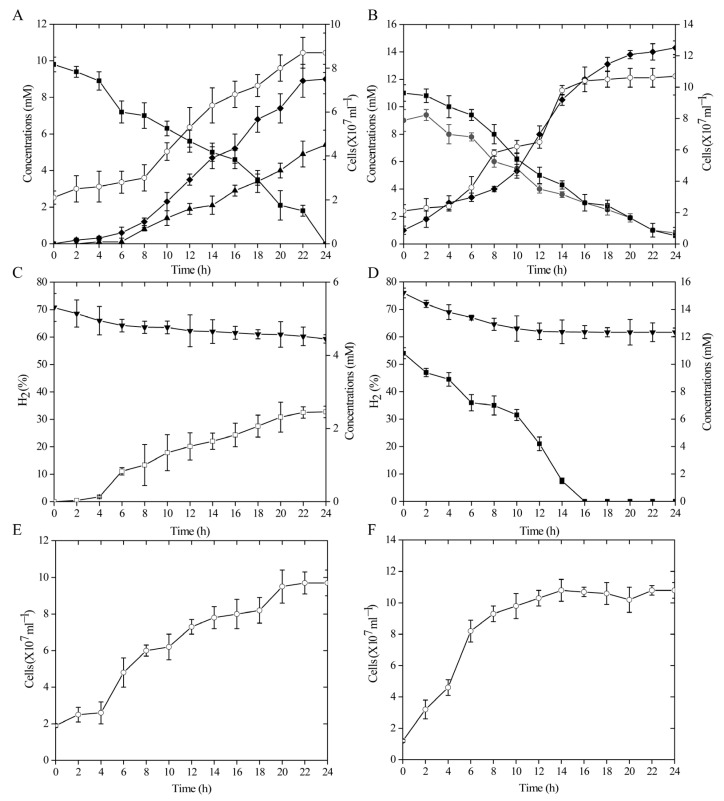
Chemolithoautotrophic growth of *S. indicum* ST-419 in the presence of S^0^ as the sole electron donor (**A**) or the sole electron acceptor (**C**). Cultures of strain ST-419 by thiosulfate oxidation (**B**) and nitrate reduction (**D**) served as controls, respectively. Cell density in S^0^ reduction and nitrate reduction is shown in (**E**,**F**). Error bars indicate the standard deviation between triplicate cultures. Closed squares, diamonds, circles, triangles and inverted triangles indicate the concentrations of NO_3_^–^, SO_4_^2−^, S_2_O_3_^2−^, N_2_ and H_2_. Open squares and circles indicate H_2_S concentration and cell numbers, respectively.

Strain ST-419 can grow chemolithoautotrophically with cyclooctasulfur or thiosulfate as the sole electron donor and nitrate as the terminal electron acceptor in an MMJS medium, as we reported previously [43]. The growth curves of strain ST-419 as well as the substrate and product concentrations over time are shown in Figure 1 (Figure 1A,B). The S^0^ granules initially floated on the medium due to the hydrophobic surface, and then gradually dipped into the medium and sank to the bottom of the bottles with the onset of cell growth and sulfur oxidation. As shown in Figure 1A,B, cultures grown with S^0^ had a significantly longer lag phase, which is necessary for cells to activate solid S^0^ prior to uptake and metabolizing. Over 24 h of incubation, elemental sulfur was gradually converted to sulfate, the final product of sulfur oxidation, reaching a maximum concentration of 8.97 mM, and accompanied by active cell growth and nitrate reduction from 8 h to 22 h until nitrate completely converted into nitrogen (Figure 1A). This reaction is congruent with the following equation: S^0^ + 1.2 NO_3_^–^ + 0.4 H_2_O→SO_4_^2–^ + 0.6 N_2_ + 0.8 H^+^ [53]. Compared to S^0^ oxidation, the overall growth was better in the thiosulfate-oxidizing culture. The growth rate of thiosulfate-grown cultures was 1.55-times higher than that of cultures grown on S^0^ (0.11 h^−1^ vs. 0.06 h^−1^), corresponding to doubling times of ~6.93 h and ~10.75 h, respectively (Figure 1A,B). The short-rod morphology and size of the cells was similar under both experimental conditions.

### 3.2. Growth Kinetics by Sulfur and Nitrate Reduction Coupled with Hydrogen Oxidization

Strain ST-419 can also grow chemolithoautotrophically with hydrogen as the sole energy source and elemental sulfur or nitrate as the sole electron acceptor [43]. Similarly, S^0^, which initially floated on the medium, gradually sank to the bottom of the bottle as cell growth and sulfur reduction began. S^0^-reducing cultures consistently grew more slowly than nitrate-reducing cultures, but similar cell densities could be achieved after 24 h (>9 × 10^7^ cells mL^−1^) (Figure 1E,F). Growth kinetics revealed that the doubling times in sulfur-reducing culture and in nitrate-reducing culture were approximately 7.70 h and 3.87 h, respectively (Figure 1E,F). The dissolved sulfide concentration reached up to 2.46 mM in cultures grown by sulfur reduction after a 24 h incubation (Figure 1C). Cell density, hydrogen consumption and sulfide production increased in parallel (Figure 1C–F). The cell morphology was also similar under both culture conditions as a short rod without obvious change in cell size.

### 3.3. General Features of Transcriptomes for S^0^ Oxidation and Reduction Conditions

**Figure 2:**
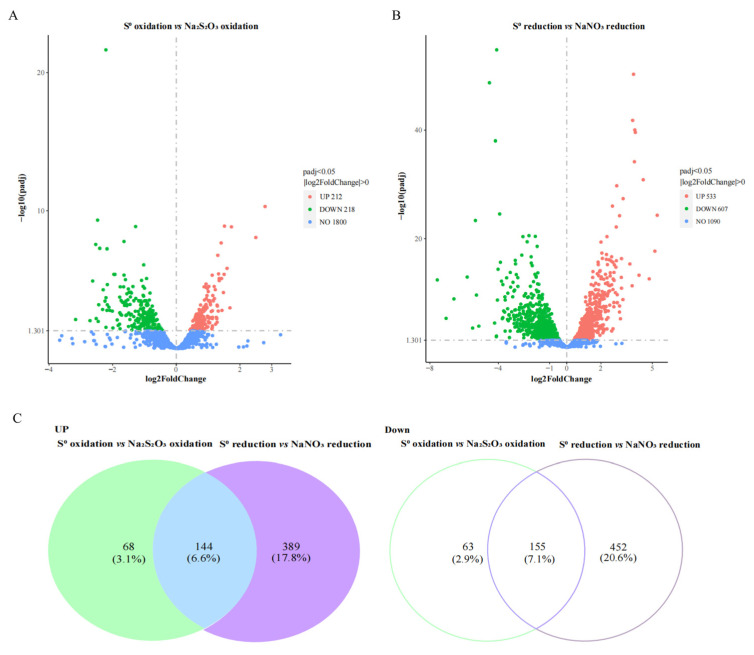
Volcano plots showing differentially expressed genes of *S. indicum* ST-419 under S^0^ oxidation (**A**) and S^0^ reduction (**B**) compared to the control. (**C**) Venn diagrams showing the differential expression gene occurring in both conditions including upregulated (Left) and downregulated (Right). % represents the proportion of differential expression genes shared in the two conditions among the CDS of the genome.

To examine changes in gene expression specifically associated with the oxidation and reduction of S^0^, we investigated the transcriptomes of strain ST-419 using cells harvested at four different conditions in two pairs: S^0^ oxidation vs. thiosulfate oxidation (S^0^ oxidation), and S^0^ reduction vs. nitrate reduction (S^0^ reduction). A principal component analysis (PCA) showed that sample variability among the experimental treatments was higher than in the biological replicates, with replicates clustering well-separated along the main axis (54.2% of total variance) (Figure S1A). The expression patterns of S^0^ oxidation and reduction transcriptomes were evaluated using a heat map, which revealed that the expression profiles were separated among S^0^ oxidation and reduction against controls samples, respectively (Figure S1B). A total of 2137 and 2140 gene transcripts were detected in the transcriptomes of strain ST-419 during oxidation of S^0^ and thiosulfate, respectively, corresponding to 97.62–97.76% of all protein coding genes. Among those transcripts, 430 differentially transcribed genes (DEGs) (*p*adj < 0.05) were identified, of which 212 were significantly increased and 218 significantly decreased (Figure 2A). Similarly, 2139 and 2250 transcripts were detected during reduction of S^0^ and nitrate, respectively (97.71–98.22% of protein coding genes), with 533 and 607 significantly increased and decreased, respectively, during reduction of S^0^ compared with nitrate reduction (Figure 2B). The list of the transcripts significantly expressed under both S^0^ oxidation and reduction is given in Table S2. Among the significant upregulation transcripts, 144 genes were shared between S^0^ oxidation and reduction, accounting for 6.6% of the total predicted coding sequences (CDS) of the genome (Figure 2C). Similarly, 155 significant downregulated transcripts were shared between S^0^ oxidation and reduction, corresponding to 7.1% of the total CDS (Figure 2C). The transcripts significantly expressed under both S^0^ oxidation and reduction in common are shown in Table S2.

### 3.4. Genes Potentially Involved in S^0^ Activation and Their Differential Expression

**Figure 3:**
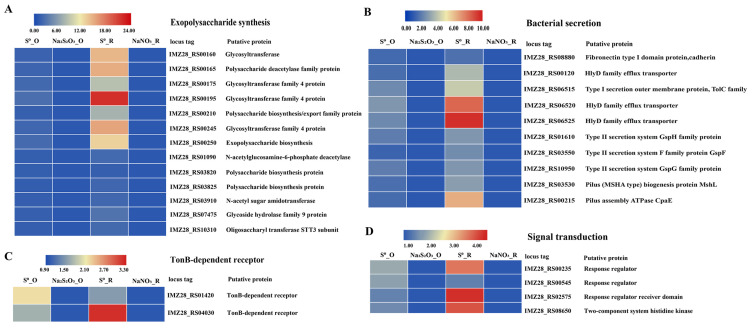
Transcriptomic analysis of genes associated with biofilm formation of strain ST-419 between S^0^ oxidation and reduction. Heat map analysis of differentially expressed genes related to exopolysaccharide synthesis (**A**), bacteria secretion (**B**), TonB-dependent receptors (**C**) and signal transduction (**D**). S^0^_O represents S^0^ oxidation; Na_2_S_2_O_3__O represents thiosulfate oxidation; S^0^_N represents S^0^ reduction and NaNO_3__N represents nitrate reduction.

Among the 144 genes significantly overexpressed in both S^0^ oxidation and S^0^ reduction, 29 gene transcripts associated with biofilm formation including exopolysaccharide synthesis, bacterial secretion, signal transduction and TonB-dependent transfer system were found (Figure 3). Among them, most of these genes were related to exopolysaccharide synthesis, including polysaccharide synthetase and glycosyltransferase, which were significantly upregulated by 1.42–2.68- and 1.53–21.79-fold in both conditions, respectively (Figure 3A). For bacterial secretion, we identified several genes encoding efflux transporters (IMZ28_RS00120, IMZ28_RS06520 and IMZ28_RS06525) belonging to the HlyD family, and the outer membrane efflux protein, TolC (IMZ28_RS06515), which were significantly upregulated by 1.65–2.83-fold and 3.85–9.64-fold in S^0^ oxidation and reduction, respectively (Figure 3B). Genes associated with type II secretion systems including GspH (IMZ28_RS01610), GspF (IMZ28_RS03550) and GspG (IMZ28_RS10950) were also significantly upregulated in S^0^ oxidation and reduction conditions (Figure 3B). The HlyD and TolC in type I secretion systems, as well as Gsp in type II secretion systems, are responsible for exporting polysaccharides to the cell surface [54,55]. In addition, a gene (IMZ28_RS08888) related to type I secretion systems, containing a cadherin tandem repeat domain, was upregulated by 1.51- and 1.77-fold under S^0^ oxidation and reduction (this difference was statistically different), respectively (Figure 3B). Two type IV pilin-related genes including a pilus assembly protein MshL (IMZ28_RS83530) and a pilus assembly ATPase CpaE (IMZ28_RS00215) were also significantly upregulated in S^0^ oxidation and reduction conditions. Cadherin-like domains and type IV pili are frequently involved in surface adhesion [56,57]. Furthermore, some of these common genes were implicated in the signal transduction, such as the two-component system and TonB-dependent receptors in the presence of S^0^ either oxidation or reduction (Figure 3C,D).

### 3.5. Genes Potentially Involved in S^0^ Uptake and Their Differential Expression

**Figure 4:**
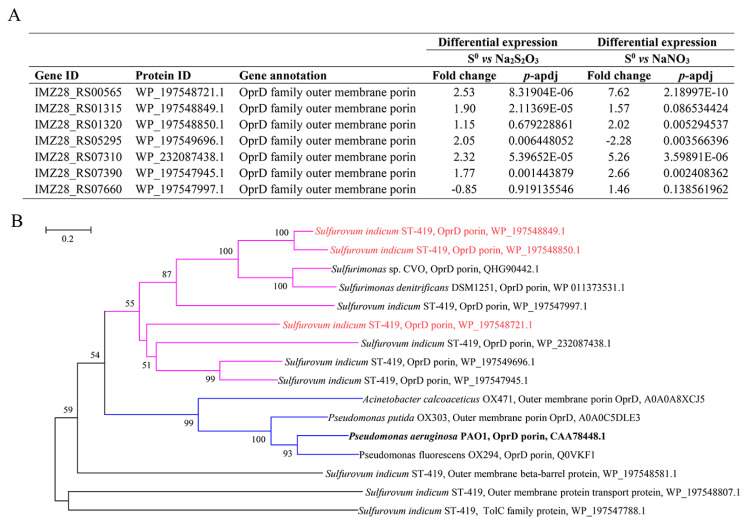
Transcriptomic analysis of genes associated with outer membrane proteins of strain ST-419 under S^0^ oxidation and reduction (**A**). Phylogenetic relationships of outer membrane protein from strain ST-419 with homologous genes in the genera of *Sulfurimonas*, *Acinetobacter* and *Pseudomonas* (**B**). The three OprD proteins in red fonts have the highest expressions under S^0^ oxidation and reduction. The OprD from *Pseudomonas aeruginosa* PAO1 in bold is the known characterized enzyme.

A total of seven genes encoding the outer membrane OprD-like porins were detected in the transcriptomic data (Figure 4A). Porins of the OprD family are diverse and allow the facilitated uptake of a variety of specific substrates [58,59], so they may be involved in the uptake and transport of sulfur into cells as detailed below. Three genes encoding porin (IMZ28_RS00565, IMZ28_RS07310 and IMZ28_RS07390) were significantly upregulated by 1.77–2.53- and 2.66–7.62-fold in S^0^ oxidation and reduction compared with the control samples, respectively (Figure 4A). Among them, the gene IMZ28_RS00565 had the highest number of transcripts with FPKM values of 9110 and 12,412 for the two conditions, respectively, except two genes expressed at much lower levels (Table S2). Furthermore, the OprD protein encoded by gene IMZ28_RS00565 shared 19% sequence identity with its homolog in *Sulfurimonas denitrificans* DSM1251 [60]. Protein domain analysis showed that the N-terminal amino acids 42-403 of IMZ28_RS00565 possessed high similarity to the major outer membrane protein OprD porin of *Campylobacterota* (pfam05538). The modeling of the structural topology revealed several potential transmembrane domains that could form a beta-barrel structure and a signal peptide, pointing to a role as an outer membrane protein [61]. However, based on the low overall similarity, a functional classification cannot be inferred at present. Additionally, two *oprD* genes (IMZ28_RS01315 and IMZ28_RS01320) were also highly expressed in S^0^ oxidation and reduction conditions. Among them, gene IMZ28_RS01315 had a higher expression level with FPKM values of 8743 and 9068 under both conditions, and was upregulated by 1.90- and 1.57-fold under S^0^ oxidation and reduction, respectively (Figure 4A; Table S2). Similarly, the other gene (IMZ28_RS01320) had the highest expression under S^0^ oxidation and reduction with FPKM values of 40,107 and 46,447, respectively, and was upregulated by 1.15- and 2.02-fold under both conditions (Figure 4A; Table S2). Furthermore, two *oprD* genes (IMZ28_RS01315 and IMZ28_RS01320) encoding proteins shared the highest amino acid sequence identity of 34% and 45% with those of *S. denitrificans* DSM1251, respectively. A phylogenetic analysis showed that they clustered together with the homologous genes of *S. denitrificans* DSM1251 and *Sulfurimonas* sp. CVO (Figure 4B), which have recently been proposed to be involved in the uptake or transport of S^0^ [60,62].

Additionally, several genes coding for transporters were differentially expressed under both S^0^ oxidation and S^0^ reduction conditions (Table S3). Among them, four genes encoding substrate binding proteins (IMZ28_RS00125, IMZ28_RS02590, IMZ28_RS09850 and IMZ28_RS09855) were detected (Table S3), which belonged to ABC-type (ATP binding cassette) transporters burning ATP to fuel substrate transport [63]. Three tripartite ATP-independent periplasmic (TRAP)-type transporters (IMZ28_RS00555 IMZ28_RS00560 and IMZ28_RS06185) were also identified (Table S3). These transporters could obtain energy to actively channel substrates from the extracellular environment to the cytoplasm by combining them with the thermodynamically favorable transport of a solute such as Na^+^ [64]. These different types of transporters are involved in the transport of various compounds, and their substrate spectrum might cover sulfur compounds. In addition, three DEGs encoding ATPase (IMZ28_RS01810, IMZ28_RS07155 and IMZ28_RS10785) were also discovered (Table S3). They could be involved in the synthesis of ATP in the process of oxidation and reduction of S^0^ by oxidative phosphorylation, when electrons released by catabolism (electrons derived from H_2_ in sulfur reduction, and from S^0^/H_2_S in sulfide oxidation) enter the respiratory chain.

### 3.6. Evaluation of the Necessity for Direct Cell-S^0^ Contact Using Dialysis Bag Incubation

**Figure 5:**
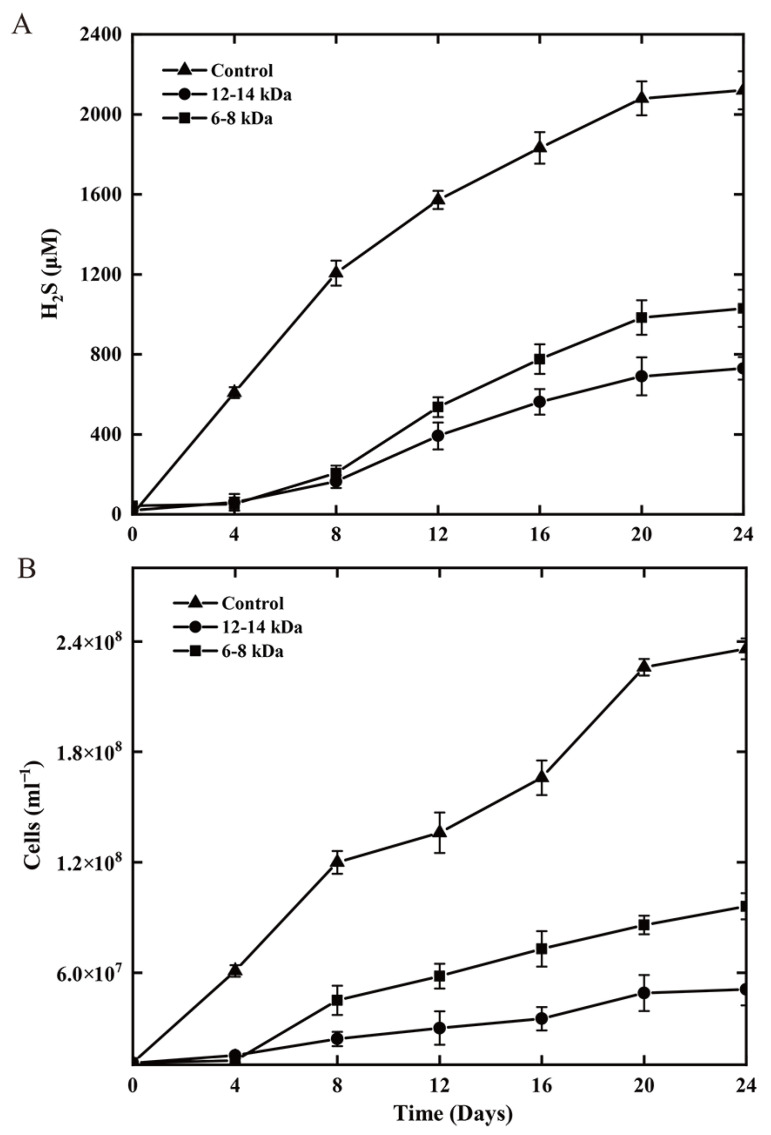
Sulfide concentrations (**A**) and cell densities (**B**) in cultures of strain ST-419 grown chemolithoautotrophically with H_2_ as the sole electron donor and S^0^ as the sole electron acceptor. S^0^ was provided in the bulk medium (control) or was sequestered in dialysis membranes (pore sizes of 6 to 8 kDa or 12 to 14 kDa) to prevent physical contact with the bulk S^0^.

To investigate whether direct physical contact between cells and sulfur granules is essential for sulfur oxidation and respiration by strain ST-419, dialysis membranes with two kinds of pore sizes (6–8 and 12–14 kDa) were used to separate cells from elemental sulfur. The results showed that direct cell contact with solid S^0^ was not required for bacterial growth and sulfide production during sulfur reduction (Figure 5). Compared to the condition without the dialysis membrane, both sulfur reduction and bacterial growth decreased to varying degrees in the presence of the dialysis bags (Figure 5). In detail, with the membranes of pore sizes 6–8 kDa and 12–14 kDa, sulfide production decreased by 49% and 34% compared to the control without membrane, respectively (Figure 5A). Correspondingly, the final cell yield decreased by 41% and 22%, respectively (Figure 5B). In contrast, no cell growth was observed in S^0^ oxidation treatments when S^0^ was sequestered in dialysis membranes. Thus, cells of the bacterium growing via S^0^ reduction did not require direct contact to S^0^ granules, whereas growing via S^0^ oxidation did require direct contact.

### 3.7. Expression of the Genes Involved in S^0^ Oxidation

**Figure 6:**
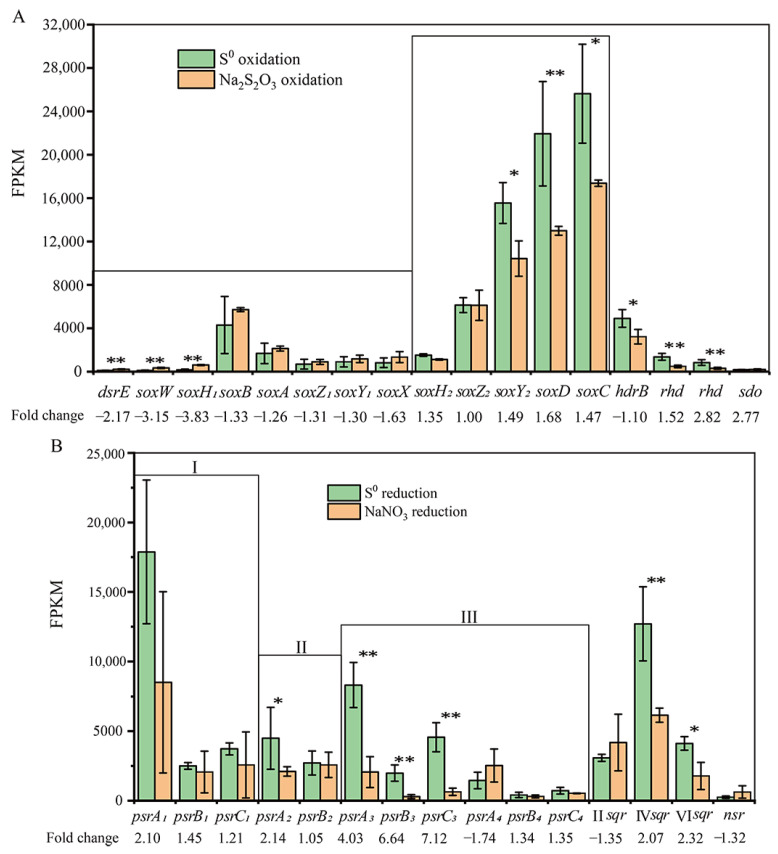
The relative abundance and expression of genes related to S^0^ oxidation (**A**) and S^0^ reduction (**B**) in strain ST-419. Light green bars show the average values of strain ST-419 grown on S^0^ oxidation and reduction, and light orange bars show the average of cultures grown on thiosulfate oxidation and nitrate reduction. Statistically significant differences are denoted by one asterisk (*p*adj < 0.05) or two asterisks (*p*adj < 0.01). The symbols of “I”, “Ⅱ” and “Ⅲ” represente the different gene cluster of polysulfide reductases. Normalized gene expression is plotted as fragments per kilobase and million reads (FPKM). Error bars indicate the standard deviation from triplicate cultures.

High transcripts of the periplasmic Sox multienzyme complex, encoded by two gene operon, *soxABXY_1_Z_1_* and *soxCDZ_2_Y_2_*, were detected, implying that strain ST-419 uses the Sox system for the oxidation of reduced sulfur compounds. However, both *sox* operons showed obvious different transcription levels between thiosulfate and S^0^ oxidation conditions (Figure 6A). The gene cluster *soxABXY_1_Z_1_* was transcribed under both growth conditions overall, but transcripts were more abundant with thiosulfate (Figure 6A). In its upstream in the genome, there are genes *dsrE* (IMZ28_RS08580), *soxW* (IMZ28_RS08585) and *soxH_1_* (IMZ28_RS08590), which were significantly upregulated by 2.17–3.83-fold under thiosulfate oxidation (Figure 6A), suggesting their involvement in this process. DsrE and SoxW are, respectively, the periplasmic and cytoplasmic thioredoxins, which take part in the electron transport process [65]. SoxH is annotated as a putative metallo-hydrolase, which is responsible for releasing sulfate from SoxY [31]. Notably, the *soxCDY_2_* genes in the second gene cluster were quite highly expressed with a FPKM value of >15,000, and significantly upregulated by 1.47–1.69-fold under S^0^ oxidation compared to thiosulfate oxidation, with the exception of the gene *soxZ_2_*, which showed no significant variation under both culture conditions (Figure 6A). In addition, a *soxH_2_*-like gene (IMZ28_RS10560) adjacent to the *soxCDY_2_Z_2_* gene cluster was expressed in raised abundance under S^0^ oxidation (Figure 6A). This indicates the important role of *soxCDY_2_Z_2_H_2_* in the oxidation of S^0^.

Among the significantly upregulated genes, three genes including *hdrB* (IMZ28_RS05750) and two genes encoding rhodanese-like proteins (IMZ28_RS02435 and IMZ28_RS02440) might also be involved in S^0^ oxidation. The gene *hdrB* (IMZ28_RS05750) was significantly upregulated by 1.52-fold under S^0^ oxidation (Figure 6A). Structural domain analysis showed that the *hdrB*-encoding protein has the cysteine functional motif, which is thought to be important for S^0^ oxidation in acidophilic sulfur oxidizers [66]. Two genes encoding rhodanese-like protein (IMZ28_RS02435 and IMZ28_RS02440) were also significantly upregulated by 2.77–2.82-fold in S^0^ oxidation (Figure 6A), which may be involved in the electron transfer during S^0^ oxidation. In addition, a gene (IMZ28_RS07900) encoding MBL-fold metallo-hydrolase showed a low expression under S^0^ oxidation (Figure 6A), although recently it was proposed to be involved in sulfur oxidation [28].

### 3.8. Expression of the Genes Involved in S^0^ Reduction

Based on genome analysis, strain ST-419 contained four types of putative polysulfide reductase-encoding genes (*psrA_1_B_1_C_1_*, *psrA_2_B_2_*, *psrA_3_B_3_C_3_* and *psrA_4_B_4_C_4_*), which may perform a sulfur reduction function. These four kinds of polysulfide reductase-encoding genes showed differential transcriptional expressions under S^0^ reduction compared to nitrate reduction (Figure 6B). Among them, the gene cluster *psrA_3_B_3_C_3_* was significantly upregulated by 4.03–7.12-fold with the high FPKM value of 1986–8312 under S^0^ reduction (Figure 6B). Three genes in the gene cluster *psrA_1_B_1_C_1_* also exhibited a high FPKM value of 2504–17,874, corresponding to an increase in gene expressions by 1.21–2.10-fold during S^0^ reduction compared to nitrate reduction (Figure 6B), although there were no significant differences between the two conditions. In contrast, the transcriptional levels of genes in *psrA_2_B_2_* and *psrA_4_B_4_C_4_* were quite low, with gene *psrA_2_* showing a significantly upregulated expression and gene *psrA_4_* showing a downregulated expression (Figure 6B). These results indicated that the polysulfide reductase encoded by *psrA_3_B_3_C_3_* may be more important for sulfur reduction.

In addition, three genes encoding for sulfide:quinone oxidoreductase (Sqr) belonging to type II, type IV and type VI, which are well known to be responsible for the oxidation of sulfide [67], showed different expression profiles during S^0^ reduction (Figure 6B). Among them, the expression of type IV *sqr* (IMZ28_RS09870) and type VI *sqr* (IMZ28_RS10500) was higher and significantly upregulated by 2.07–2.32-fold during S^0^ reduction (Figure 6B), implying their certain role in the process of sulfur reduction. The involvement of Sqr towards the direction of the reduction of sulfur compounds has been shown in *Sulfurovum* sp. NBC37-1 and in the thermophilic bacterium *Thermovibrio ammonificans* [68,69], which oxidizes the quinone pool and contributes to the reduction of elemental sulfur. In addition, the gene encoding NADH-dependent sulfur reductase (Nsr, IMZ28_RS07000), which has been proposed to be involved in sulfur reduction in *T. ammonificans* [69], showed a low expression level and was downregulated during S^0^ reduction (Figure 6B).

### 3.9. qRT-PCR Analyses of Sulfur Oxidation and Reduction Gene Transcripts

In order to verify the differential transcription of the genes putatively involved in sulfur metabolism, we performed qRT-PCR on 15 selected genes encoding: the beta lactamase precursor *soxH_1_* (IMZ28_RS08590), the thiosulfohydrolase *soxB* (IMZ28_RS08600), the sulfur oxidation c-type cytochrome *soxA* (IMZ28_RS08605), the thiosulfate oxidation carrier complex protein *soxZ_1_* (IMZ28_RS08610), the thiosulfate oxidation carrier protein *soxY_1_* (IMZ28_RS08615), the sulfur oxidation c-type cytochrome *soxX* (IMZ28_RS08620), the beta-lactamase domain protein *soxH_2_* (IMZ28_RS10560), the thiosulfate oxidation carrier protein *soxZ_2_* (IMZ28_RS10570), the c-type cytochrome *soxY_2_* (IMZ28_RS10575), the sulfite dehydrogenase *soxD* (IMZ28_RS10580), the sulfur oxidation molybdopterin C protein *soxC* (IMZ28_RS10585), the molybdopterin oxidoreductase *psrA_1_* (IMZ28_RS08440), the molybdopterin oxidoreductase *psrA_2_* (IMZ28_RS03620), the molybdopterin oxidoreductase *psrA_3_* (IMZ28_RS06785) and the molybdopterin oxidoreductase *psrA_4_* (IMZ28_RS07170) (Table S1). qRT-PCR results indicated that the transcripts for *soxH_1_*, *soxB*, *soxA*, *soxZ_1_*, *soxY_1_* and *soxX* were higher in thiosulfate oxidation than S^0^ oxidation, and among them, the expressions of *soxH_1_* and *soxB* were significantly upregulated by 3.31- and 1.47-fold under thiosulfate oxidation (Figure S3A). The expression levels of *soxH_2_*, *soxZ_2_*, *soxY_2_*, *soxD* and *soxC* were greater upon S^0^ oxidation than thiosulfate oxidation. Among them, genes of *soxY_2_*, *soxD* and *soxC* were upregulated by 1.49-, 1.58- and 1.67-fold, respectively, under S^0^ oxidation as compared to thiosulfate oxidation (Figure S3A). Furthermore, qRT-PCR quantification of the Psr large-subunit-encoding genes (*psrA_1_*, *psrA_2_*, *psrA_3_* and *psrA_4_*) revealed that only *psrA_3_* was significantly upregulated by 3.94-fold in S^0^ reduction versus nitrate reduction, whereas *psrA_1_* and *psrA_2_* showed a weak upregulation and *psrA_4_* showed a downregulation (Figure S3B). These results indicate that the expression patterns from RNA-seq analysis are reliable.

### 3.10. Phylogenetic and Sequence Analyses of Diverse Polysulfide Reductases

**Figure 7:**
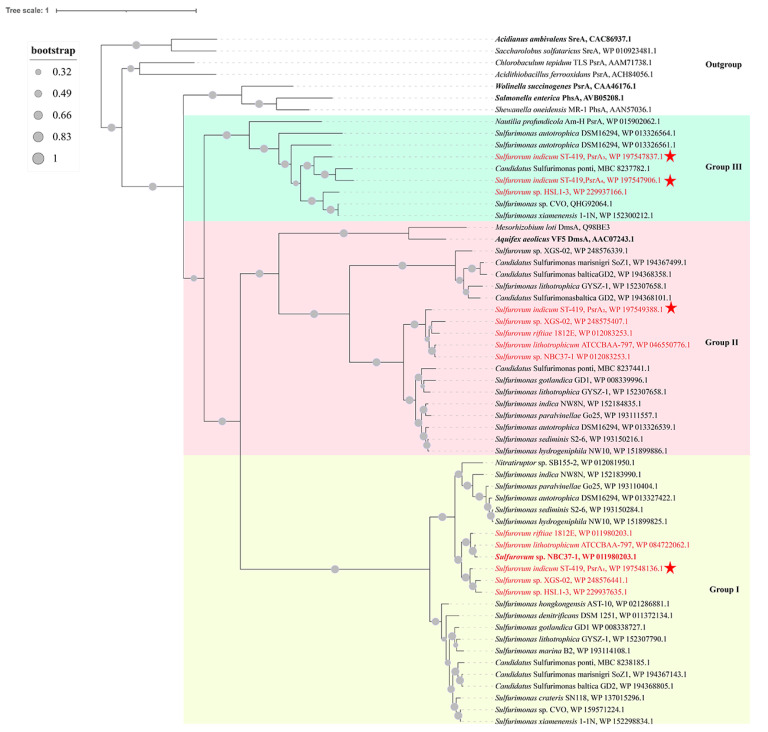
Phylogenetic relationships of putative PsrAs from strain ST-419 with various sequences of the sulfur reductase/polysulfide reductase/thiosulfate reductase family. The polysulfide reductase present in cultured strains of the genus *Sulfurovum* are shown in red and the homologous genes in strain ST-419 are marked with an asterisk. Biochemically characterized enzymes are shown in bold. The red stars represented the polysulfide reductases of strain ST-419. Amino acid sequences were derived from the non-redundant protein database of NCBI and accession numbers are shown in the rear. Bootstrap values based on 1000 replicates are shown at branch nodes. Bar = 1.0 substitutions per protein position.

The phylogenetic analysis based on deduced amino acid sequences encoded by four *psrA*-like genes showed that they formed three separate clades (group I, group II and group III) (Figure 7). Furthermore, they shared a common ancestor with the PsrA of *W. succinogenes*, the thiosulfate reductase PhsA of *Salmonella enterica* and the sulfur reductase SreA of *A*. *ambivalens* (Figure 7), which have been experimentally demonstrated to reduce sulfur or polysulfide [70,71,72]. The PsrA_1_ in group I is present in all *Sulfurovum* species and formed a monophyletic cluster, suggesting this group might be conserved within genus *Sulfurovum* (Figure 7). The genes in *psrA_1_B_1_C_1_* shared the highest sequence similarity of 81–89% with the known sulfur respiration enzymes PsrABC of *Sulfurovum* sp. NBC37-1 [67]. The PsrA_2_ from genus *Sulfurovum* clustered into one group, and clustered with its homologs from the genus *Sulfurimonas* and the sulfur reductase SreA from *Aquifex aeolicus* (Figure 7). The gene cluster *psrA_2_B_2_* shared 57–68% sequence similarity with the homologs from *Sulfurimonas hydrogeniphila* NW10, which was recently proposed to be involved in sulfur reduction [34]. As for group III, phylogenetic analysis indicated that PsrA_3_ and PsrA_4_ of strain ST-419 clustered with the homologous gene from *Sulfurovum* sp. HSL1-3, and furthermore formed one branch with other polysulfide reductase from the genera *Sulfurimonas* and *Nautilia* (Figure 7). The gene cluster of *psrA_3_B_3_C_3_* and *psrA_4_B_4_C_4_* shared 20–65% sequence similarity, and both had the highest similarity of 15–46% and 13–40%, respectively, with the PsrABC from *W. succinogenes* [70]. Furthermore, we detected the distribution of the *psr* gene cluster in genus *Sulfurovum* from 5fivepure isolated genomes and 122 metagenomes currently available on NCBI database. The result revealed that the four *psr* genotypes were present in *Sulfurovum* spp. from various environmental niches, including hydrothermal vents, terrestrial biofilm, hydrocarbon-rich habitats and marine sediments (Table S4). This provides evidence that the genotype of strain ST-419 is shared by other *Sulfurovum* spp., and suggests that this mechanism of sulfur reduction may be environmentally significant.

The primary amino acid sequences of the four PsrAs from *S. indicum* ST-419 were aligned with those of homologous enzymes that were biochemically characterized and shown to reduce S^0^ to H_2_S in vitro (Figure S4). Sequence comparison showed that several important cysteine residues are conserved at different positions, as indicated by one or two asterisks on the Figure S4. Among them, a conserved Cys176 residue was found to be necessary for the sulfur reductase activity in *A. aeolicus* [73]; this residue is assumed to be redox-active and involved in a Cys-S intermediate during the catalytic cycle. This conserved cysteine was also found in the PsrA of *W. succinogenes* (Cys-173) and the PhsA of *S. enterica* (Cys-178), which is responsible for sulfur reduction [34,73]. In the genome of strain ST-419, the Cys residue is conserved in the four PsrA proteins at positions 171, 247, 183 and 195, respectively (two asterisks, Figure S4). This conserved cysteine is also detected in the PsrA of *Sulfurovum* sp. NBC37-1 (Cys-257) (Figure S4). Overall, these observations strongly suggest that the Psr-like enzymes might be directly involved in S^0^ reduction in strain ST-419. Furthermore, none of the four PsrA proteins contained a typical twin arginine motif or a signal peptide, suggesting that they probably work in the cytoplasm.

## 4. Discussion

Elemental sulfur is abundant in hydrothermal vents, and its associated catabolism by the dominant chemolithoautotrophic *Campylobacterota* remains poorly understood. The genus *Sulfurovum* has been confirmed as one of the most predominant members of deep-sea hydrothermal prokaryotic residents that support the unique chemoautotrophic ecosystem [37], and is strongly involved in the oxidation of reduced inorganic sulfur compounds [26,38,74,75]. In the present study, we investigated the mechanisms of cyclooctasulfur activation and metabolisms when it is used as an electron donor or acceptor during sulfur oxidation and reduction in the deep-sea hydrothermal vent bacterium *S. indicum*. To our knowledge, this is the first report about the activation and metabolism mechanisms associated with S^0^ utilization in the genus *Sulfurovum*.

Considering the extremely low solubility and reactivity of S^0^ [76,77], microorganisms most likely need a specific activation or solubilization mechanism to make S^0^ available for their energy metabolism. Biofilm formation is an important way for cells to adsorb S^0^ in some bacteria and archaea, as previously observed [78,79,80,81]. During this process, flagella play an important role in initial biofilm development, which have been suggested to mediate attachment to sulfur particles in acidophilic and neutrophilic sulfur-oxidizing bacteria, including *Sulfurimonas* of the phylum *Campylobacterota* [18,60,82,83]. In this study, obvious biofilms were also observed on the surface of S^0^ particles by SEM (Figure S2). However, unlike the previously studied bacteria, strain ST-419 and other members of *Sulfurovum* entirely lacked the genes for surface-associated flagellar proteins and the bacterial chemotactic system (Figure S5). In contrast, genes related to biofilm formation including those involved in exopolysaccharide synthesis, bacteria secretion, signal transduction and the TonB-dependent transfer system were significantly upregulated in the presence of S^0^ (Figure 3). It is well-known that exopolysaccharides are one of the main components of biofilms and play a key role in the initial bacterial attachment [84,85]. The bacterial secretion systems of type I (such as HlyD and TolC family proteins) and type II were also markedly overexpressed, with the possible effect of facilitating surface adhesion, as suggested by another study [78]. Additionally, the genes involved in signal transduction and TonB-dependent receptors, which are essential for biofilm formation, were also clearly induced in the presence of S^0^ (Figure 3C,D). Therefore, biofilm formation by *Sulfurovum* may be a crucial step in activating S^0^ for both sulfur oxidation and reduction as supported by morphological and transcriptomic data.

The high expressions of frequently observed outer membrane proteins (OMP) in cells oxidizing and reducing solid S^0^ (Figure 4) suggest their involvement in utilizing this substrate. Two *oprD*-like porin genes (IMZ28_RS01315 and IMZ28_RS01320) were highly expressed under S^0^ oxidation and reduction (Figure 4), and they shared more than 30% sequence identity with the homologous genes in *S. denitrificans* DSM 1251 and *Sulfurimonas* sp. CVO, which were shown to be involved in the utilization of solid S^0^ by a transcriptomic and/or proteomic analyses [60,62]. Furthermore, in contrast to what was found in the genus *Sulfurimonas*, we found another highly expressed gene *oprD* (IMZ28_RS00565) in strain ST-419, which showed a 2.53- and 7.63-fold increase under both conditions, respectively (Figure 4; Table S2). All three proteins possessed the conserved domain of the OprD family, which is likely to play an important role in *Campylobacterota* as the homologs are frequently found in the genomes of strains in this phylum. Thus, we proposed that these porins may play a key role in S^0^ uptake in *Campylobacterota*. In addition, in acidophilic sulfur-oxidizing bacteria, some outer membrane proteins, such as Omp40 and Omp44, are also considered to play a role in sulfur attachment and transportation [17,86,87]. However, the true membrane proteins involved in S^0^ transport have not yet been experimentally confirmed in either acidophilic or neutrophilic sulfur-oxidizing bacteria [17,60,62]. Thus, further research is needed to determine whether these outer membrane proteins are directly involved in S^0^ degradation/activation, or whether they facilitate S^0^ transport into cells.

**Figure 8:**
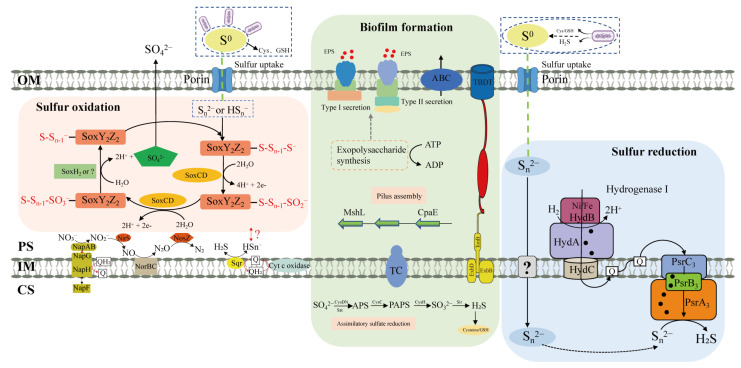
Proposed model for extracellular S^0^ activation, transportation and intracellular oxidation and reduction in strain ST-419. OM, outer membrane; PS, periplasmic space; IM, inner membrane; CS, cytoplasmic space; Sox, sulfur-oxidation-multienzyme complex; Q, quinone molecules; Psr, polysulfide reductase; TC, two-component system; TBDT, TonB-dependent transporters. Hypothetical reactions concerning energy conservation are indicated by a question mark.

In addition to the direct contact of cells to insoluble S^0^, a non-contact or cooperative mechanism (coexistence of contact and non-contact activation) could be involved in S^0^ activation. In this study, we found that strain ST-419 growing via S^0^ reduction did not require direct contact to S^0^, whereas cells growing via S^0^ oxidation did require direct access (Figure 5), which is consistent with our previous reports in other species of *Sulfurovum* and *Sulfurimonas* [34,75]. Thus, a mechanism of activation of S^0^ without cell contact also likely exists. Furthermore, we found some genes associated with cysteine and glutathione synthesis which were differentially upregulated under S^0^ oxidation and reduction (Table S5). Under S^0^ oxidation conditions, sulfur activation requires a direct cell contact, as cellular access to bulk sulfur contributes to the efficiency of the overall process by keeping cells and their substrate in close proximity, and avoiding the oxidation of the -SH-group-containing compounds (Figure 8). Under S^0^ reduction conditions, the sulfide produced by the metabolism, and/or the release of compounds containing -SH groups such as cysteine, GSH [88,89], could be involved in the activation of sulfur through a nucleophilic attack (Figure 8). However, in natural environments, contact and non-contact activation mechanisms can always coexist, in a way we propose naming cooperative activation, which is to some extent similar to the metal oxidation mechanism observed in acidophilic sulfur-oxidizing bacteria [20,21,90]. In the cooperative activation, the bacterial cells would continuously release the compounds containing a -SH group to catalyze the transition of the S^0^ ring to the -S-S^−^ group. Continuous release would lead the bacteria to consume substantial amounts of the high-energy substrate. To minimize substrate consumption, the bacteria would get as close as possible to the elemental sulfur surface. Thus, we propose that the cells should be in contact with S^0^ before the release of the reducing reagents. However, it is still not known which reducing substances the bacterium releases, and this question is expected to be solved by transcriptome analysis of dialysis bags or by metabolomics.

Transcriptome analysis of cultures growing by sulfur oxidation showed that the *soxABXY_1_Z_1_* gene cluster was more expressed under thiosulfate oxidation condition, while the *soxCDY_2_Z_2_* gene cluster was more expressed under S^0^ oxidation conditions (Figure 6A). Consistently, these two *sox* gene clusters were also differentially regulated by different sulfur compounds in *Allochromatium vinosum* and *S. denitrificans* [6,60,91,92]. It is possible that S^0^ oxidation in strain ST-419 is performed solely by the gene cluster *soxCDY_2_Z_2_*. Indeed, *Sulfurimonas* sp. CVO from an oil field is able to oxidize S^0^ to thiosulfate and sulfate with only *soxCDY_2_Z_2_* in the absence of *soxABXY_1_Z_1_* [62]. Thus, we propose that the S^0^ oxidation pathway is performed by *Sulfurovum* species as follows (Figure 8). First, circular S_8_ is transformed into linear polysulfide (S*_n_*^-^, HS*_n_*^–^) in a currently unknown way, and then is transported into the cellular periplasm by a transporter, possibly via an OprD-like porin. In the periplasmic space, polysulfide is covalently bound to a cysteine residue of SoxY_2_, and generates a thiocysteine-S sulfate residue (SoxZ_2_Y_2_-S-S_7_-S^-^). The outer sulfone sulfur of the cystein-persulfide on SoxY_2_ is then oxidized by SoxCD to form a cysteine-S sulfate residue (SoxZ_2_Y_2_-S-S_7_-SO_2_^-^), and subsequently oxidized to form SoxZ_2_Y_2_-S-S_7_-SO_3_^2-^. Finally, this complex is hydrolyzed to sulfate by SoxH_2_ or by another way, and regenerates the SoxZ_2_Y_2_ complex (SoxZ_2_Y_2_-SH) (Figure 8). At present, we are not able to resolve how the sulfonate group bound to SoxY_2_Z_2_ is hydrolyzed to form sulfate; possibly by the putative periplasmic metallo-hydrolase encoded by IMZ28_RS10560. This scenario seems plausible as this gene is located next to *soxCDY_2_Z_2_* and its homologs can be retrieved in many other sulfur-oxidizing *Campylobacterota* with conserved synteny as a thiol hydrolase. Another possibility might be that SoxCDY_2_Z_2_ can catalyze the reaction by itself, as previously supposed in *S. denitrificans* DSM1251 [60], though there is no relevant experimental evidence.

During sulfur reduction, four polysulfide reductases of three groups including group I (*psrA_1_B_1_C_1_*), group II (*psrA_2_B_2_*) and group III (*psrA_3_B_3_C_3_* and *psrA_4_B_4_C_4_*) were differentially expressed (Figure 6B and Figure 7). Protein domain analysis showed that all these proteins were located in the cytoplasm, implying that *S. indicum* performed a cytoplasmic sulfur reduction. Among them, the transcript of *psrA_3_B_3_C_3_* was highly abundant and significantly upregulated, implying its essential role in sulfur reduction. This is significantly different from our recent report of a *Sulfurimonas* isolate, which used both periplasmic and cytoplasmic polysulfide reductases, encoded by genes *psrA_1_B_1_CDE* and *psrA_2_B_2_*, respectively, to perform cyclooctasulfur reduction [34]. Together with the results in *Sulfurimonas* spp., cytoplasmic sulfur reduction seems to be a crucial catabolic pathway in the phylum *Campylobacterota*. The overall mechanism proposed for sulfur respiration in strain ST-419 is summarized in Figure 8. The reduction of elemental sulfur could be conducted by an electron transport chain from molecular hydrogen, via the upregulated membrane-bound hydrogenase, with menaquinone (MK) as electron carriers in the membrane, to reduce polysulfide from producing H_2_S by PsrA_3_B_3_C_3_ (Figure 8). The end product of H_2_S then diffuses outside the cell and helps convert bulk sulfur to dissolved polysulfide. The reduction of sulfur and HS- diffusion out of the cell allows the formation of a proton gradient. However, at this point, it is difficult to explain the coexistence of four different cytoplasmic polysulfide reductases. We speculate that these polysulfide reductases may facilitate the host adaptation to variations in the dynamic environments of hydrothermal vents, which needs further investigations.

## 5. Conclusions

In this report, we investigated the processes of activation, uptake and subsequent oxidation and reduction of cyclooctasulfur in *Sulfurovum indicum*, a neutrophilic chemolithoautotrophic bacterium of the phylum *Campylobacterota* that is predominant in deep-sea hydrothermal ecosystems. We described the key genes and metabolic pathways involved in biofilm formation, sulfur uptake, periplasmic sulfur oxidation and cytoplasmic sulfur reduction, coupled with nitrate reduction and hydrogen oxidation, respectively. Our isolate and other *Sulfurovum* genomes entirely lack the genes for surface-associated flagellar proteins and bacterial chemotactic systems. We propose that a cooperative mechanism may exist for S^0^ activation, which would involve the reducing compounds such as Cys, GSH and H_2_S. Transcriptomic data indicated that the complex *soxCDY_2_Z_2_H*_2_ plays a role in S^0^ oxidization in the periplasm, while among the four polysulfide reductases, *psrA_3_B_3_C_3_* plays a more important role in sulfur reduction in cytoplasm. These mechanisms may be applicable to other *Campylobacterota*. The results of this study provide a better understanding of how cells derive energy from elemental sulfur, which is abundant in the current and past marine ecosystem. In the future, further genetic and biochemical investigations will be needed to confirm the genes involved in S^0^ activation, and to validate the tentatively proposed models for sulfur oxidation and reduction.

## Data Availability

Data is contained within the article and Supplementary Material.

## Supplementary Materials

The following supporting information can be downloaded at: https://www.mdpi.com/article/10.3390/antiox12030627/s1, Figure S1: Principal component analysis (PCA) and heatmap analysis of RNA-seq transcriptome data demonstrated S^0^-utilized gene expression patterns. A PCA plot of all RNA-seq samples showing how samples subjected to different treatments clustered separately (A). A heatmap including the gene cluster analysis done with log10 (FPKM+1) values (B); Figure S2: Scanning electron micrographs of S^0^ particles collected from cultures of strain ST-419 grown by S^0^ oxidation (B) and by S^0^ reduction (D). Smooth sulfur granules from uninoculated media was the control (A,C); Figure S3: qRT-PCR analyses of the expression of genes encoding S^0^ oxidation (A) and S^0^ reduction (B) of strain ST-419; Figure S4: Multiple sequence alignment of the region containing the proposed molybdenum ligand from the catalytic subunit of enzymes similar to polysulfide/sulfur/thiosulfate reductases; Figure S5: The clusters of orthologous genes (COGs) in the genome of strain ST-419. Table S1: Primers for qRT-PCR used in this study; Table S2: he differentially up-regulated and down-regulated transcripts in S^0^ oxidation and reduction compared to the controls, as well as the common genes including up and down regulated in both conditions; Table S3: Differential expression of genes involved in transport and ATPase under S^0^ oxidation and reduction; Table S4: Distribution of genes encoding polysulfide reductase in the genomes of cultured isolates and metagenome-assembled genomes (MAGs) of the genus *Sulfurovum*; Table S5: Differential expression of genes involved in cysteine and glutathione synthesis under S^0^ oxidation and reduction.
