# Supplementary material for: Transcriptome Analysis of Cyclooctasulfur Oxidation and Reduction by the Neutrophilic Chemolithoautotrophic Sulfurovum indicum from Deep-Sea Hydrothermal Ecosystems
Source: Antioxidants (Basel). 2023 Mar 3;12(3):627. doi: 10.3390/antiox12030627 (PMC10045233; doi:10.3390/antiox12030627)
Supplement: Supplementary file 1 [file antioxidants-12-00627-s001.zip › antioxidants-2202666-supplementary.pdf]

Experimental procedures

MMJS medium composition

MMJS medium consisted of NaCl (30 g l^{-1}), NH_4Cl (0.25 g l^{-1}), KCl (0.33 g l^{-1}), $CaCl_2 \cdot 2H_2O$ (0.14 g l^{-1}), $MgCl_2 \cdot 6H_2O$ (4.18 g l^{-1}), K_2HPO_4 (0.14 g l^{-1}), $NaHCO_3$ (1 g l^{-1}), $Na_2S_2O_3 \cdot 5H_2O$ (10 mM), Wolfe's vitamins (1 ml l^{-1}) and trace element solution (10 ml l^{-1}) under a gas phase mixture of 80% N_2/20% CO_2 (200 kPa), which is always used for the enrichment and isolation of chemoautotrophic sulfur-oxidizing bacteria (Takai *et al.*, 2003).

Transcriptional profiling

Library preparation for strand-specific transcriptome sequencing

A total amount of 3 μg RNA per sample was used as input material for RNA sample preparations. Sequencing libraries were generated using the NEBNext® Ultra™ Directional RNA Library Prep Kit for Illumina® (NEB, USA) following manufacturer's recommendations and index codes were added to attribute sequences to each sample. rRNA is removed using a specialized kit that leaves the mRNA. Fragmentation was carried out using divalent cations under elevated temperature in First Strand Synthesis Reaction Buffer (5X). First strand cDNA was synthesized using random hexamer primer and M-MuLV Reverse Transcriptase (RNaseH-). Second strand cDNA synthesis was subsequently performed using DNA Polymerase I and RNase H. In the reaction buffer, dNTPs with dTTP were replaced by dUTP. Remaining overhangs were converted into blunt ends *via* exonuclease/polymerase activities. After adenylation of 3' ends of DNA fragments, adaptors with hairpin loop structure were ligated to prepare for hybridization. In order to select cDNA fragments of preferentially 370-420 bp in length, the library fragments were purified with AMPure XP system (Beckman Coulter, Beverly, USA). Then 3 μL USER Enzyme (NEB，USA) was used with size-selected, adaptor-ligated cDNA at 37 °C for 15 min followed by 5 min at 95 °C before PCR. Then PCR was performed with Phusion High-Fidelity DNA polymerase, Universal PCR primers and Index Primer. Products were finally purified (AMPure XP system) and library quality was assessed on an Agilent Bioanalyzer 2100 system.

Clustering and sequencing

The clustering of the index-coded samples was performed on a cBot Cluster Generation System using TruSeq PE Cluster Kit v3-cBot-HS (Illumia) according to the manufacturer's instructions.

After cluster generation, the library preparations were sequenced on an Illumina Hiseq platform and paired-end reads were generated.

Data Analysis

Raw data (raw reads) of fastq format were firstly processed through in-house perl scripts. In this step, clean data (clean reads) were obtained by removing adaptor-containing reads, poly-conteining reads and low-quality reads from the raw data. At the same time, Q20, Q30 and GC content the clean data were calculated. All the downstream analyses were based on the clean data with high quality. Reference genome and gene model annotation files were downloaded from genome website directly. Both building index of reference genome and aligning clean reads to reference genome were used Bowtie2-2.2.3. HTSeq v0.6.1 was used to count the reads numbers mapped to each gene. And then FPKM of each gene was calculated based on the length of the gene and reads count mapped to this gene. FPKM, expected number of Fragments Per Kilobase of transcript sequence per Millions base pairs sequenced, considers the effect of sequencing depth and gene length for the reads count at the same time, and is currently one of the most commonly used method forestimating gene expression levels.

Differential expression analysis

Differential expression analysis of two catabolic incubation conditions (three biological replicates per condition) was performed using the DESeq R package (1.20.0). DESeq provides standard statistical values for determining differential expression in digital gene expression data using a model based on the negative binomial distribution. The resulting *p*-values were adjusted using the Benjamini and Hochberg's approach to control for false discovery rate. Genes with an adjusted p-value<0.05 found by DESeq were assigned as differentially expressed. Prior to differential gene expression analysis, for each sequenced library, the read counts were adjusted by edgeR program package through one scaling normalized factor. Differential expression analysis of two conditions was performed using the DEGSeq R package (1.20.0). Corrected *p*adj of 0.05 and log2 (Fold change) of 0 were set as the threshold for a significant differential expression.

Reference:

Takai, K.; Inagaki, F.; Nakagawa, S.; Hirayama, H.; Nunoura, T.; Sako, Y.; Nealson, K.H.; Horikoshi, K. Isolation and phylogenetic diversity of members of previously uncultivated epsilon-Proteobacteria in deep- sea hydrothermal ffelds. *FEMS. Microbiol. Lett.* **2003**, 218,167-174.

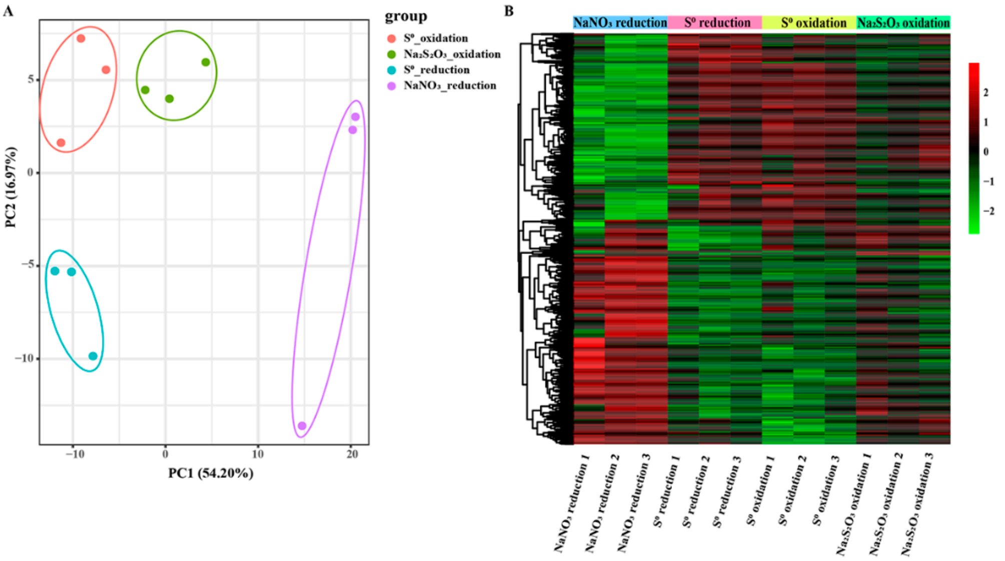

Figure S1 Principal component analysis (PCA) and heatmap analysis of RNA-seq transcriptome data demonstrated S^0-utilized gene expression patterns. A PCA plot of all RNA-seq samples showing how samples subjected to different treatments clustered separately (A). A heatmap including the gene cluster analysis done with log10 (FPKM+1) values (B). Red colour represents highly expressed genes, while blue colour represents lowly expressed genes. The color annotation bar on the far left represents a group of genes with similar transcription trends.

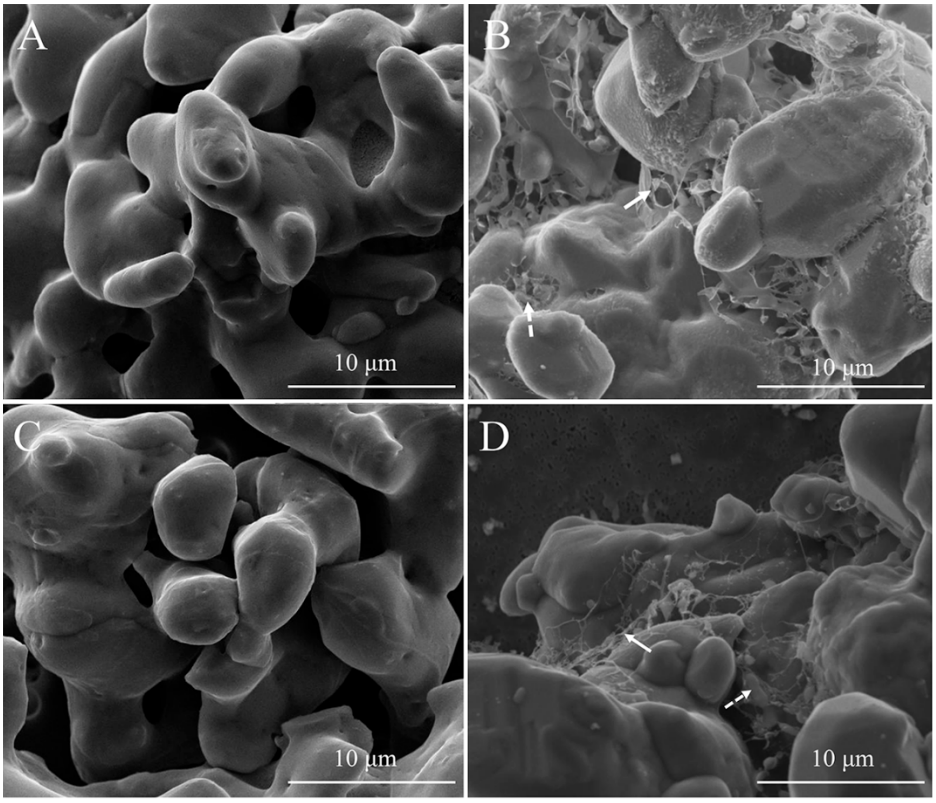

Figure S2 Scanning electron micrographs of S^0 particles collected from cultures of strain ST-419 grown by S^0 oxidation (B) and by S^0 reduction (D). Smooth sulfur granules from uninoculated media was the control (A, C). The arrows indicate biofilm covered (solid line) or bacterial cells (dashed line).

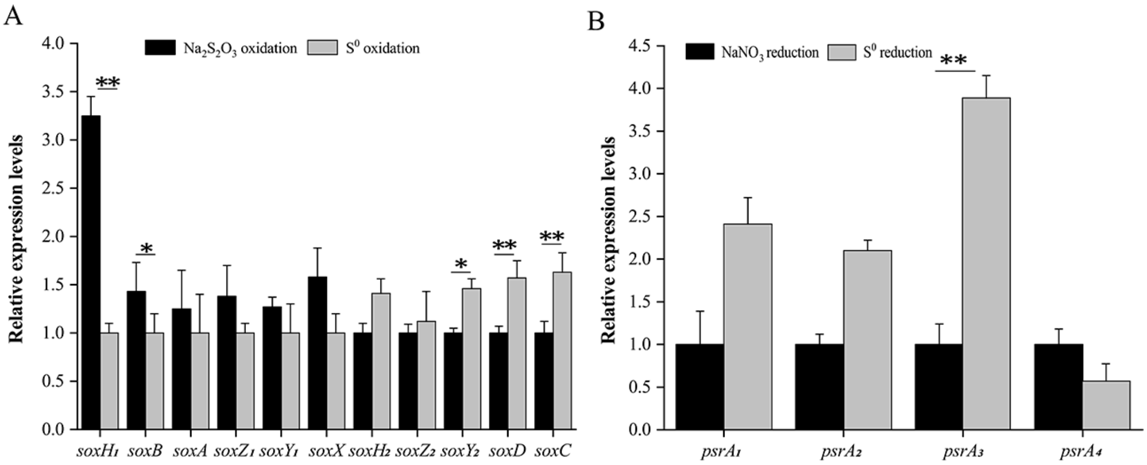

Figure S3 qRT-PCR analyses of the expression of genes encoding S^0 oxidation (A) and S^0 reduction (B) of strain ST-419. * represents $p < 0.05$ and ** represents $p < 0.01$. Error bars indicate the standard deviation between triplicate cultures.

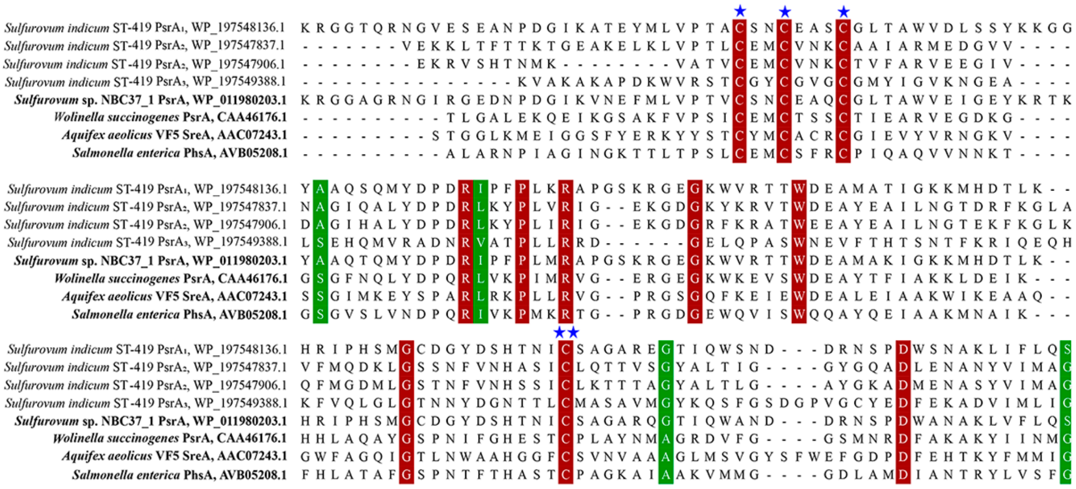

Figure S4 Multiple sequence alignment of the region containing the proposed molybdenum ligand from the catalytic subunit of enzymes similar to polysulfide/sulfur/thiosulfate reductases. Identical and similar amino acid residues are highlighted in red and green, respectively. Biochemically characterized enzymes are indicated in bold. PsrA is the catalytic subunit of polysulfide reductase from *W. succinogenes* and *Sulfurovum* sp. NBC37-1; SreA is the catalytic subunit of sulfur reductase from *A. aeolicus*; PhsA is the catalytic subunit of thiosulfate reductase from *S. enterica*.

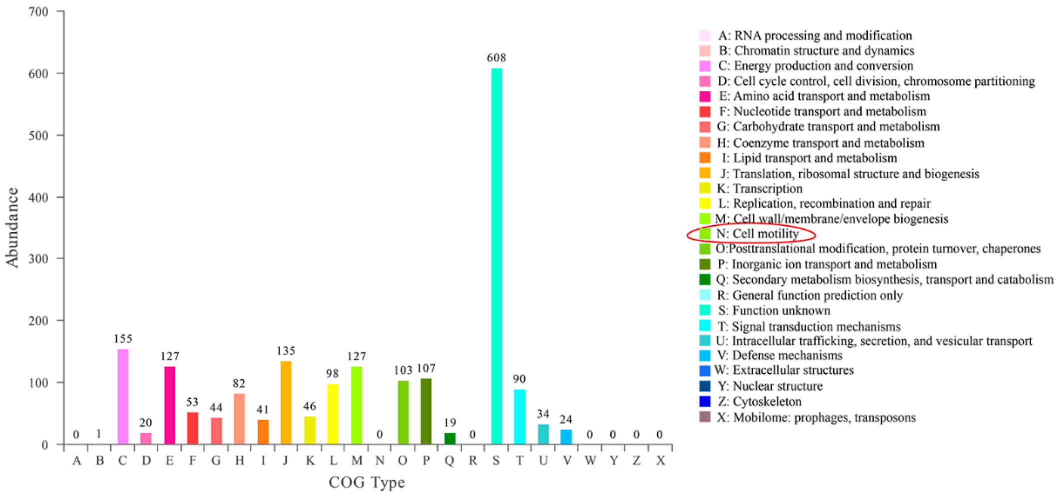

Figure S5 The clusters of orthologous genes (COGs) in the genome of strain ST-419.

Table S1 Primers for qRT-PCR used in this study.

Table S2 The differentially up-regulated and down-regulated transcripts in S^0 oxidation and reduction compared to the controls, as well as the common genes including up and down regulated in both conditions.

Table S3 Differential expression of genes involved in transport and ATPase under S^0 oxidation and reduction.

Table S4 Distribution of genes encoding polysulfide reductase in the genomes of cultured isolates and metagenome-assembled genomes (MAGs) of the genus *Sulfurovum*.

Table S5 Differential expression of genes involved in cysteine and glutathione synthesis under S^0 oxidation and reduction.
