# Supplementary material for: Transcriptome Analysis of Cyclooctasulfur Oxidation and Reduction by the Neutrophilic Chemolithoautotrophic Sulfurovum indicum from Deep-Sea Hydrothermal Ecosystems
Source: Antioxidants (Basel). 2023 Mar 3;12(3):627. doi: 10.3390/antiox12030627 (PMC10045233; doi:10.3390/antiox12030627)
Supplement: Supplementary file 1 [file antioxidants-12-00627-s001.zip › Table S1.pdf]

Table S1 Primers for qRT-PCR used in this study.

Gene name	Gene ID	Primer name	Prime sequence	Product size (bp)	Amplification efficiency (%)	Slope
soxH_1	IMZ28_RS08590	*sox*H_1-F	5'-AGTTGTCTCTCTGGCTGCATTTGAC-3'	118	99.91	–3.324
		soxH_1-R	5'-GACCATATTCCCGCCGTTTTGTTTG-3'			
soxB	IMZ28_RS08600	*soxB*-F	5'-TCGCAAACCTGATGGAAGACATAGC-3'	81	99.87	–3.325
		soxB-R	5'-AACGACTCATATCACCACCCTGTTG-3'			
soxA	IMZ28_RS08605	*soxA*-F	5'-TGAAGAGGAGTTGGGCGGAGATG-3'	141	98.99	–3.346
		soxA-R	5'-GCCTGCATCACCTGATCCAATCC-3'			
soxZ_1	IMZ28_RS08610	*sox*Z_1-F	5'-AGGTATGGGAAACAGTTTCGAC-3'	106	99.66	–3.330
		soxZ_1-R	5'-TTCTTCTCGCCTGTGTTGTC-3'			
soxY_1	IMZ28_RS08615	*sox*Y_1-F	5'-GAAGCGATCACGGGCGGTAAAG-3'	85	99.87	–3.325
		soxY_1-R	5'-CAACTGCTCCGTTCTCTGCGATC-3'			
soxX	IMZ28_RS08620	*soxX*-F	5'-AACAGATGCGGAGGCGATTGC-3'	121	99.90	–3.324
		soxX-R	5'-ACCGTCTTTGCCTTTCTGTTCAATG-3'			
soxH_2	IMZ28_RS10560	*sox*H_2-F	5'-GGCAGGTGAGGCGGAGAAATG-3'	92	98.78	–3.352
		soxH_2-R	5'-AGGCTCTTGTCGGATAGGCAAATTC-3'			
soxZ_2	IMZ28_RS10575	*sox*Z_2-F	5'-CATGCTTACTTACGACCAGGCAAAG-3'	142	98.86	–3.350
		soxZ_2-R	5'-CTTGAATTTCCACTGCGGGTTCTTG-3'			
soxY_2	IMZ28_RS10580	*sox*Y_2-F	5'-CCTGATGCCTGGACAGCACATAC-3'	118	99.80	–3.327
		soxY_2-R	5'-CTCCTCCGTTACTTGCCACCTTTG-3'			
soxD	IMZ28_RS10585	*soxD*-F	5'-TGTTGTCGGCAGTTGTTGTAGCG-3'	116	99.96	–3.323
		soxD-R	5'-CACAGGATATGTCACACCACCATCG-3'			
soxC	IMZ28_RS10590	*soxC*-F	5'-ATCAGGTAAAGCGATCCAGCACTTC-3'	87	99.92	–3.324
		soxC-R	5'-AGATCCGTCCAAGGCTTCTCAGG-3'			

psrA$_1$	IMZ28_RS08440	*psrA*$_1$-F	5’-AGCAACACAGCCGTCAAGATGG-3’	149	98.89	–3.349
		psrA$_1$-R	5’-CTACCTCTTCCCAAATCTCGCCAAG-3’			
psrA$_2$	IMZ28_RS03620	*psrA*$_2$-F	5’-CCAGATGGTACGTGCGGACAAC-3’	129	99.60	–3.332
		psrA$_2$-R	5’-TGCTGTTCCTGGATACGCTTGAATG-3’			
psrA$_3$	IMZ28_RS06785	*psrA*$_3$-F	5’-GGCGATCTTGAATGGTACGGACAG-3’	91	99.87	–3.325
		psrA$_3$-R	5’-GGCACAGTAACCGATAGTGGAACG-3’			
psrA$_4$	IMZ28_RS07170	*psrA*$_4$-F	5’-AACAGAACCCCATGCAATCCATACC-3’	128	99.93	–3.324
		psrA$_4$-R	5’-CGGCAGCACGACATCAGACATC-3’			
16S rRNA		16S rRNA-F	5’-CTACCAAGCCAATGACGCCTAGC-3’	94	99.99	–3.322
		16S rRNA-R	5’-CCCACTGCTGCCTCCCGTAG-3’			
