# Supplementary figures and images for: Transcriptome Analysis of Cyclooctasulfur Oxidation and Reduction by the Neutrophilic Chemolithoautotrophic Sulfurovum indicum from Deep-Sea Hydrothermal Ecosystems
Source: Antioxidants (Basel). 2023 Mar 3;12(3):627. doi: 10.3390/antiox12030627 (PMC10045233; doi:10.3390/antiox12030627)

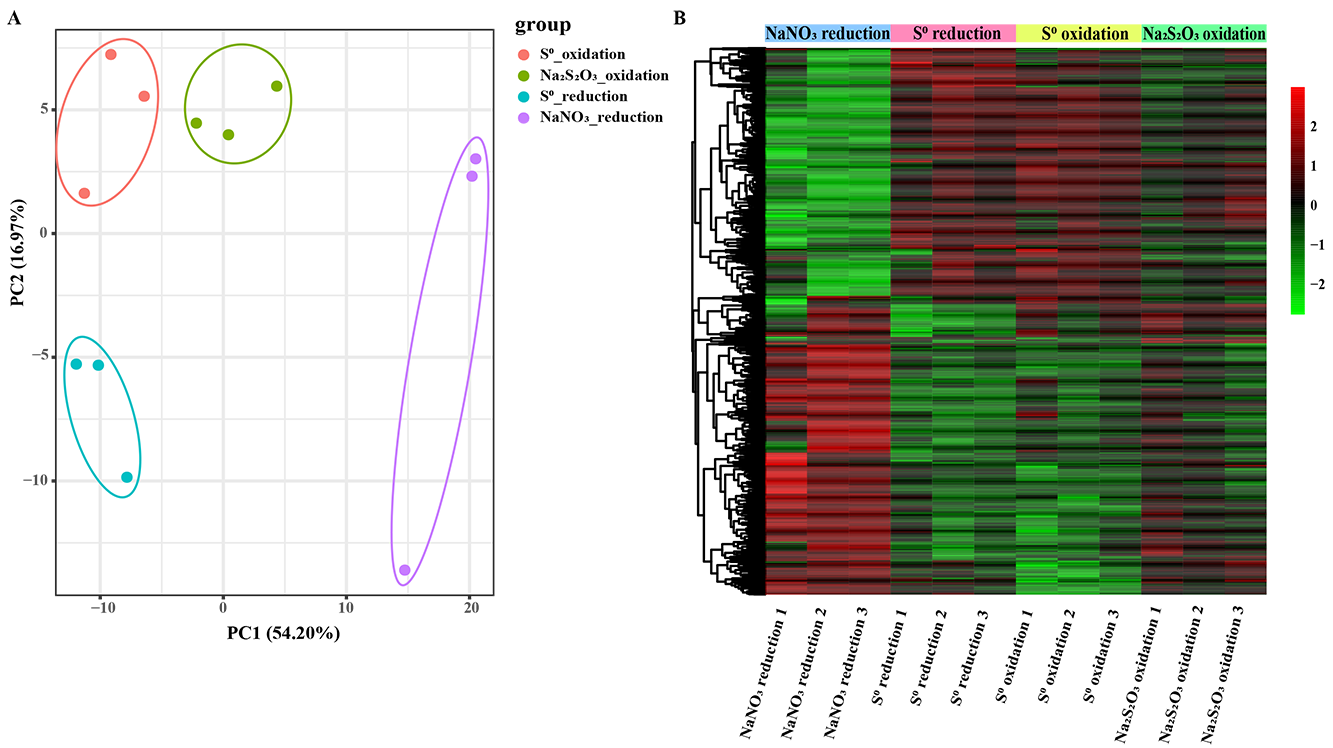

Supplement: Supplementary file 1 [file antioxidants-12-00627-s001.zip › Figure S1.tif]

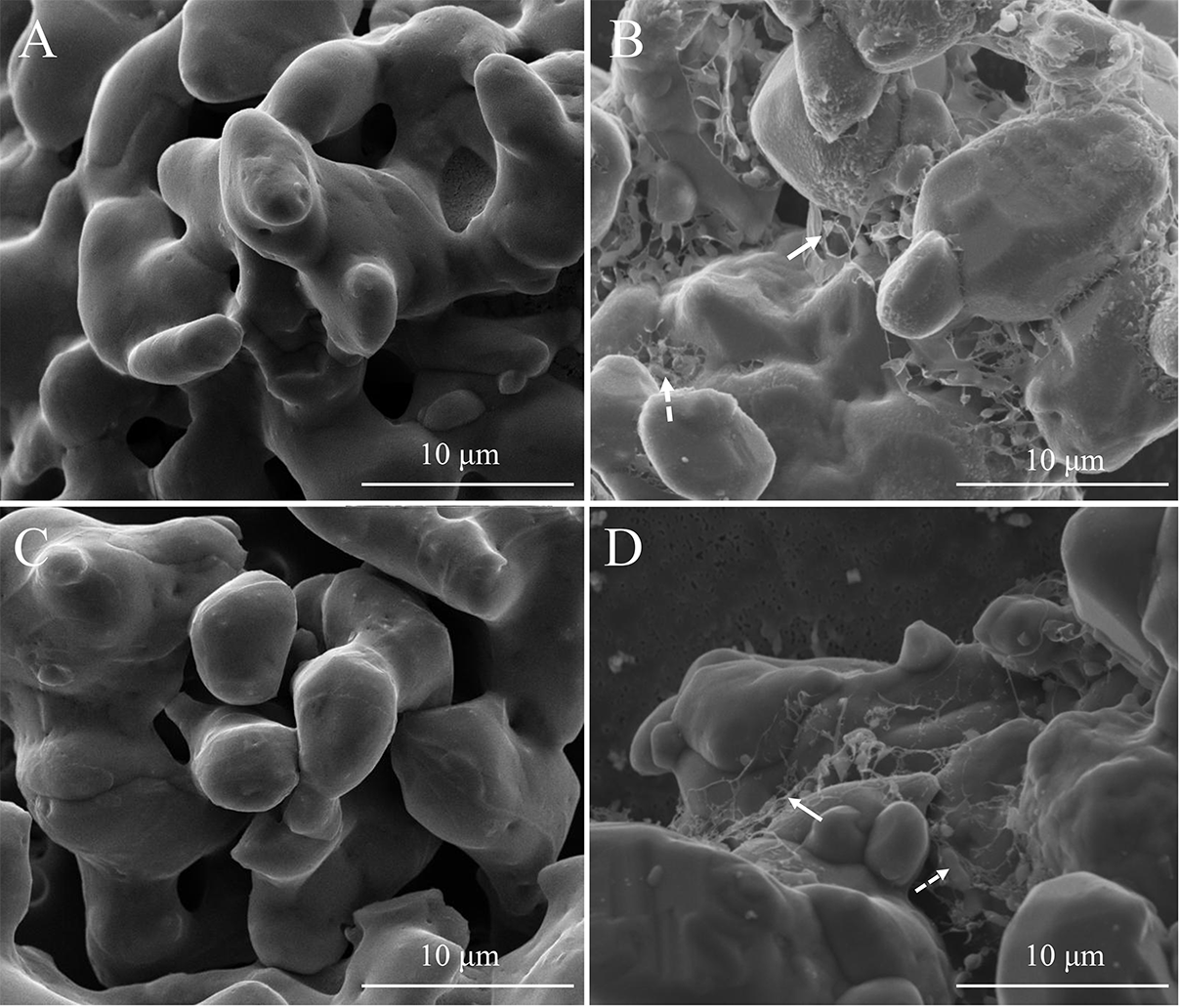

Supplement: Supplementary file 1 [file antioxidants-12-00627-s001.zip › Figure S2.tif]

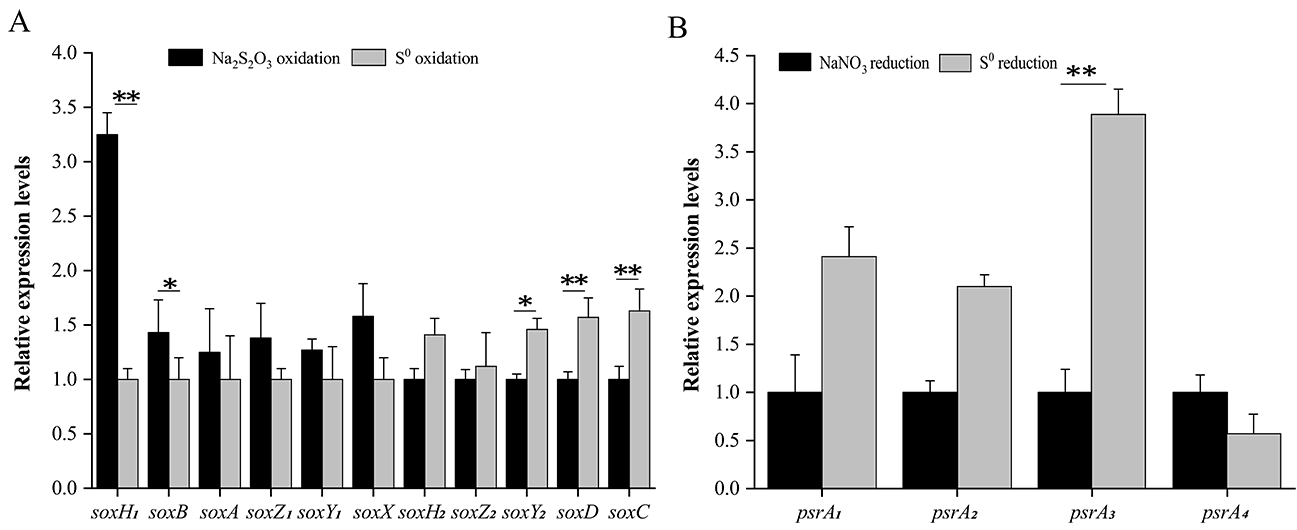

Supplement: Supplementary file 1 [file antioxidants-12-00627-s001.zip › Figure S3.tif]

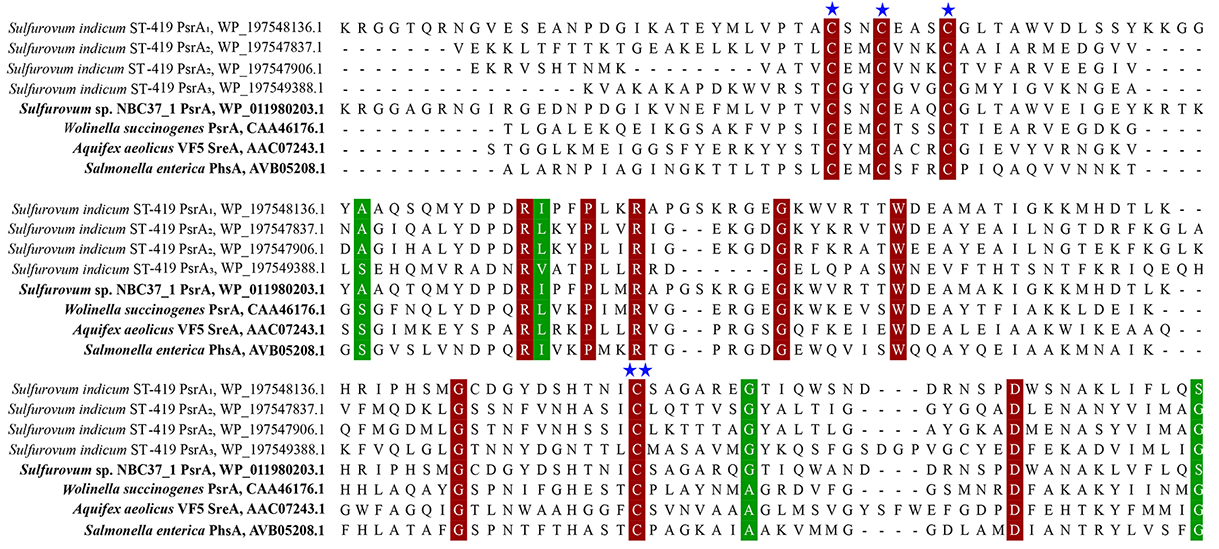

Supplement: Supplementary file 1 [file antioxidants-12-00627-s001.zip › Figure S4.tif]

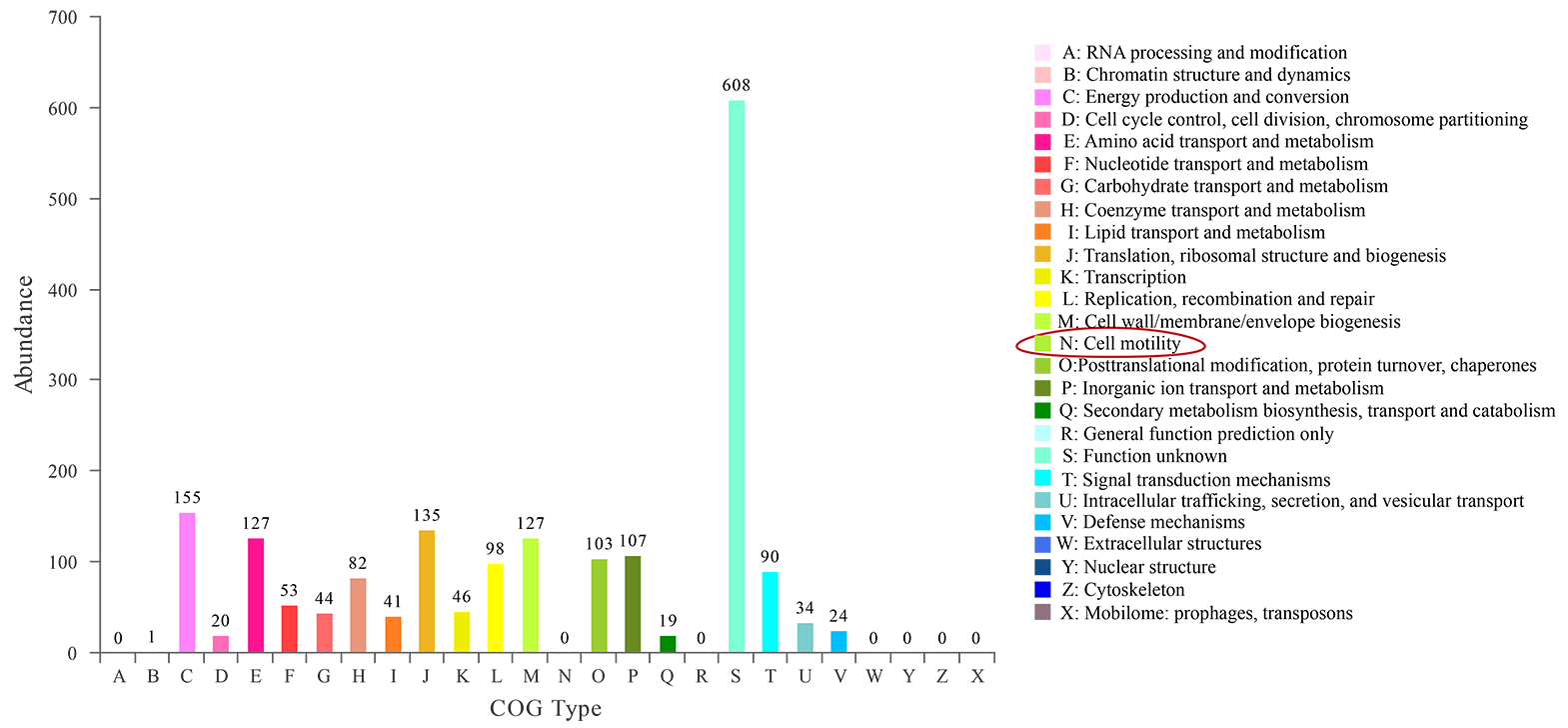

Supplement: Supplementary file 1 [file antioxidants-12-00627-s001.zip › Figure S5.tif]
